# Collagen XV preserves heart function and protects from pathological remodelling after myocardial infarction

**DOI:** 10.1111/febs.70212

**Published:** 2025-08-20

**Authors:** Sanna‐Maria Karppinen, Miki Aho, Zoltan Szabo, Johanna Magga, Laura Vainio, Erhe Gao, Paul Janmey, Lauri Eklund, Karolina Rasi, Ilkka Miinalainen, Lynn Y. Sakai, Lasse Pakanen, Heikki Huikuri, Juhani Junttila, Risto Kerkelä, Taina Pihlajaniemi

**Affiliations:** ^1^ ECM‐Hypoxia Research Unit, Faculty of Biochemistry and Molecular Medicine University of Oulu Oulu Finland; ^2^ Molecular Cardiology Group, Research Unit of Biomedicine and Internal Medicine University of Oulu Oulu Finland; ^3^ Biocenter Oulu University of Oulu Oulu Finland; ^4^ Aging and Cardiovascular Discovery Center, Lewis Katz School of Medicine Temple University Philadelphia Pennsylvania USA; ^5^ Department of Physiology, Perelman School of Medicine University of Pennsylvania Philadelphia Pennsylvania USA; ^6^ Department of Oncology and Radiotherapy Oulu University Hospital Oulu Finland; ^7^ Department of Molecular & Medical Genetics Oregon Health & Science University Portland Oregon USA; ^8^ Forensic Medicine Unit Finnish Institute for Health and Welfare Oulu Finland; ^9^ Department of Forensic Medicine, Research Unit of Biomedicine and Internal Medicine, Medical Research Center Oulu University of Oulu Oulu Finland; ^10^ Cardiology Research Group, Research Unit of Biomedicine and Internal Medicine University of Oulu/Oulu University Hospital Oulu Finland

**Keywords:** collagen XV, extracellular matrix remodelling, fibrosis, infarct scar, tissue stiffness

## Abstract

Increasing knowledge of the components involved in left ventricle (LV) remodelling and fibrotic processes after a myocardial infarction is crucial to understanding heart pathology. We have here analysed collagen XV (ColXV) expression in human myocardial infarct samples and assessed how its deficiency affects cardiac responses, such as fibrogenesis and tissue stiffness, after acute myocardial infarction (AMI) in mice. We first observed high ColXV expression in human infarction scars. After ligating the left anterior descending artery in mice, cardiac function and remodelling were monitored by echocardiography, elasticity assessment, immunohistochemical analysis and ultrastructural assessments. After AMI, *Col15a1* ^*−/−*^ mice showed significantly increased tissue stiffness and upregulation of fibrosis‐related genes in the remote myocardium. Striking differences were observed between the genotypes in the scar ultrastructure, protein compositions, cardiomyocyte morphology and intracellular architecture. Furthermore, the proportion of immature collagen fibres in the infarct border zone increased in *Col15a1* ^*−/−*^ mice, suggesting fragility and poor scar resistance to mechanical stress. Structural parameters indicated more substantial LV remodelling in the knockout mice, leading to a more dilated ventricle. Functionally, the ejection fraction and fractional shortening decreased significantly in *Col15a1* ^*−/−*^ mice, indicating impaired heart contractile capacity. The results show that in the event of an AMI, ColXV plays an essential role in sustaining cardiac structure and function. In the absence of ColXV, dysregulated remodelling results in disrupted scar and infarct border zone, and stiffer left ventricle. These changes lead to a more severe cardiac phenotype and may affect long‐term survival after AMI.

## Graphical abstract

We analysed collagen XV expression in myocardial infarct samples and assessed how its deficiency affects cardiac responses, such as fibrogenesis and tissue stiffness. In mice, damage caused by myocardial infarction is exacerbated in the absence of collagen XV and leads to pathological remodelling and more severe left ventricle dysfunction (left panel) compared with the wild‐type litter mates. These changes are accompanied by wide‐ranging effects, including phenotypical (middle panel) and molecular (right panel) changes in the infarct scar area, infarct border zone, and remote left ventricle. These changes lead to a more severe cardiac phenotype and may affect long‐term survival. Image created with BioRender.

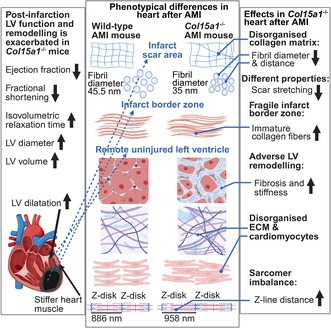

**Abbreviations**
BM: basement membrane
ColXV: collagen XV
ECM: extracellular matrix
EF: ejection fraction
FAC: fractional area change
FC: fold change
FS: fractional shortening
KO: knockout
LAD: left anterior descending
LV: left ventricular
SD: standard deviation
WT: wild‐type

## Introduction

An intact and properly organised extracellular matrix (ECM) is required for appropriate heart homeostasis. Several progressive heart defects, which are caused, for example, by hypertension or ischemia, are associated with defects in the ECM proteins and the structure, amount and quality of the connective tissue [1, 2]. The ECM plays a crucial role in cardiac remodelling after myocardial infarction and in maintaining the morphofunctional properties of the cardiac tissue. The ECM serves to support and couple cardiomyocytes, preserve ventricular geometry, limit excessive strain, prevent sarcomeric deformation and mediate mechanical signals. However, the specific functions of many ECM components in these processes are not clear.

After myocardial infarction (MI), the left ventricle undergoes a remodelling process that includes geometrical, biomechanical and biochemical changes (e.g. dilatation, fibrosis and increased tissue stiffness) due to the loss of contracting cardiomyocytes [3]. Following MI, there is initial infarct expansion occurring within hours of cardiomyocyte injury, resulting in wall thinning and ventricular dilatation. During the early phase after MI, the ventricular dilation is also augmented by cardiomyocyte lengthening and thinning through lateral slippage of muscle fibres [4]. In addition, to stabilise the contractile function, cardiomyocyte hypertrophy is induced as an early compensatory response [3]. The late phase of post‐MI remodelling involves the remote nonischemic myocardium, and is characterised by cardiomyocyte hypertrophy and left ventricular dilatation. Besides the myocytes, cardiac fibroblasts, inflammatory cells and capillary microcirculation, the ECM is also a critical component of the left ventricular (LV) remodelling and tissue repair, including two different fibrotic responses [5]. The first response, which is the formation of a fibrotic scar, is called replacement or reparative fibrosis and is an important process in preventing the ventricular wall rupture [6]. In the second response, reactive fibrosis is induced in the infarct border zone and in the remote myocardium, outside the scar area, by increased mechanical stress due to initial tissue damage and other factors. In fact, heart failure is most often caused by remodelling of the noninfarcted part of the LV. This condition leads to altered chamber compliance, stiffening of the tissue and increased fibrosis, progressively impairing cardiac function and eventually causing heart failure. Emerging data indicate that the physiological level of cardiac stiffness ensures not only the cardiomyocyte functionality but also the overall diastolic function of the ventricles [1, 2, 3]. Additionally, changes in cardiomyocyte morphology and fibrillar collagen organisation, which are processes also highly influenced by the ECM, have been linked to diastolic dysfunction and heart failure [1, 2].

Collagen XV (ColXV) is an evolutionarily conserved nonfibrillar collagen [7]. Although not a structural part of basement membranes (BMs), ColXV is located in close proximity to them and has been proposed as an anchoring link between BMs and the fibrillar collagens of the surrounding connective tissue [8, 9]. Dysregulation of ColXV is associated with different fibrotic diseases [7]. ColXV is upregulated in human fibrotic kidney and liver fibrosis in the mouse model of cholestatic liver disease, as well as in advanced stages of fibrosis in humans and rats [10, 11, 12]. On the contrary, it is downregulated in an inherited connective tissue disease, Dupuytren's contracture, which is characterised by progressive fibrosis of the palmar fascia [13]. Lack of ColXV in mice (*Col15a1* ^*−/−*^) results in skeletal myopathy and a cardiac phenotype that resembles human dilated cardiomyopathy (DCM) in certain aspects [14, 15].

However, the roles of ColXV in response to internal cardiac stress, either with respect to fibrosis or cellular properties, are unknown. Here we showed that ColXV is expressed in human infarction scars and subsequently explored its involvement in key postinfarction remodelling processes using the left anterior descending (LAD) artery ligation model in mice. The results indicate that the lack of ColXV after AMI causes changes in ECM and cellular properties leading to fibrosis and stiffer cardiac tissue. This contributes not only to the pathological changes of the infarction scar but also to the border zone and the remote LV, which together affect the deterioration of cardiac function.

## Results

### ColXV expression was increased in the human infarcted myocardium

**Fig. 1:**
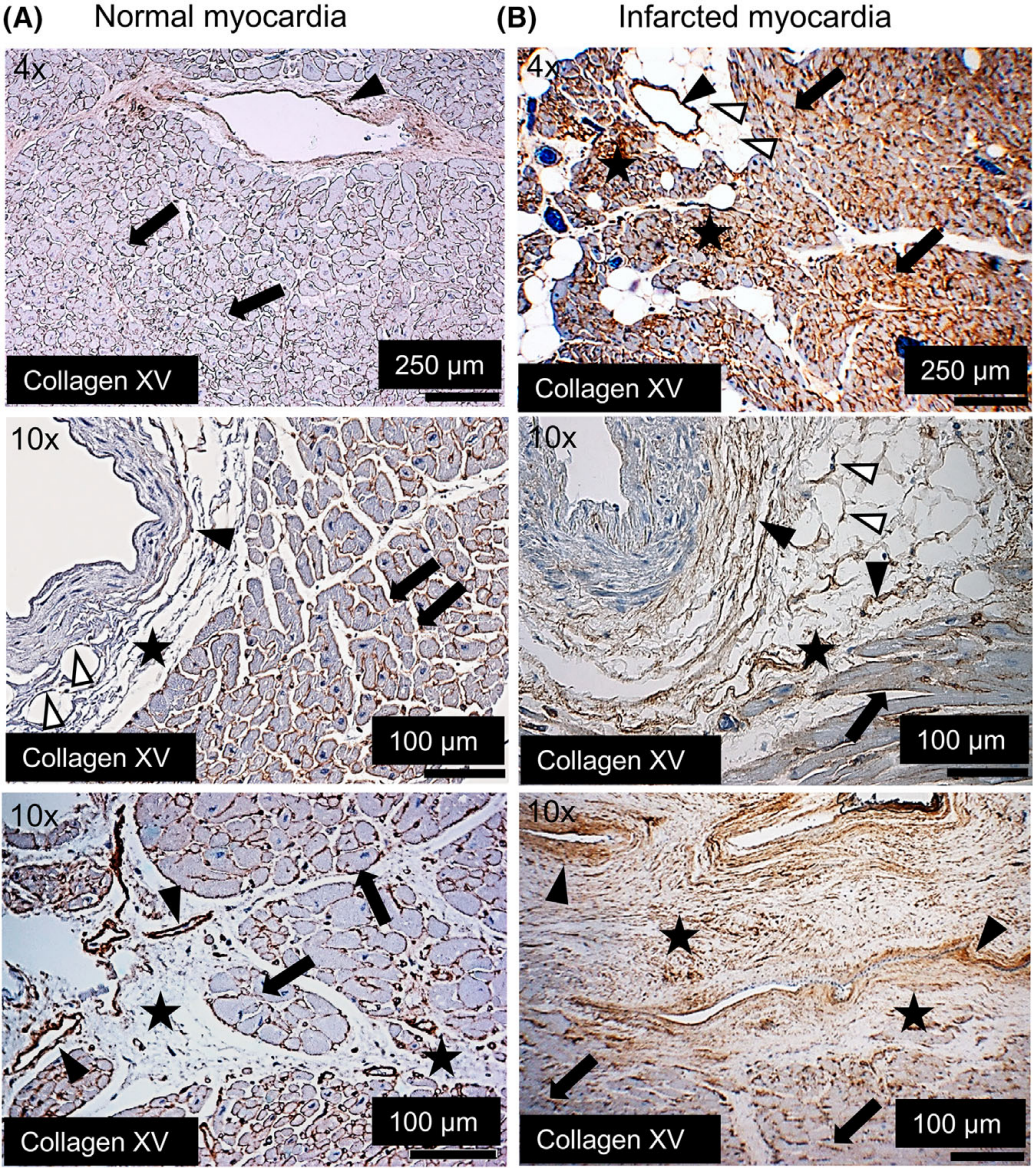
Expression and localisation of collagen XV in normal and infarcted human myocardia. Representative immunohistochemical figures of (A) a normal human myocardia from control individuals (*n* = 5) and (B) an infarcted myocardia (*n* = 20 individuals) showing ColXV upregulation in an infarcted myocardia. ColXV localises to the BM zones of the vascular structures (black arrowheads), adipocytes (white arrowheads) and cardiomyocytes (arrows), and is upregulated in the interstitial matrix and the areas of perivascular fibrosis (stars) in the infarcted samples. Original magnifications were 4× and 10×.

Our earlier observation of cardiac phenotype in mice lacking ColXV and the possible involvement of this collagen in fibrotic processes induced us to study its localisation and expression in human MI [15]. Autopsy myocardium samples from individuals who died from noncardiac causes and from AMI were analysed using immunohistochemistry. In the control samples, ColXV was located in the BM zones of the capillaries, adipocytes and cardiomyocytes (Fig. 1A) representing low staining category (relative amount of signal < 30%). Interestingly, the immunohistochemical signal of ColXV showed an increase in infarct samples, representing medium staining category (relative amount of signal between 30% and 60%) in the noninfarcted myocardium and high staining category (relative amount of signal > 60%) in the scar area (representative images shown in Fig. 1B), suggesting the role of this collagen in the postinfarcted myocardium. An increase in the ColXV signal was observed, especially in areas containing fibrosis and fibroblasts, in the areas of perivascular fibrosis around the tunica adventitia of large coronary vessels. In addition to the BM zones, the ColXV signal was present in the interstitial matrix of the infarcted myocardium.

### In a normal heart, ColXV is primarily expressed by endothelial cells and fibroblasts

**Fig. 2:**
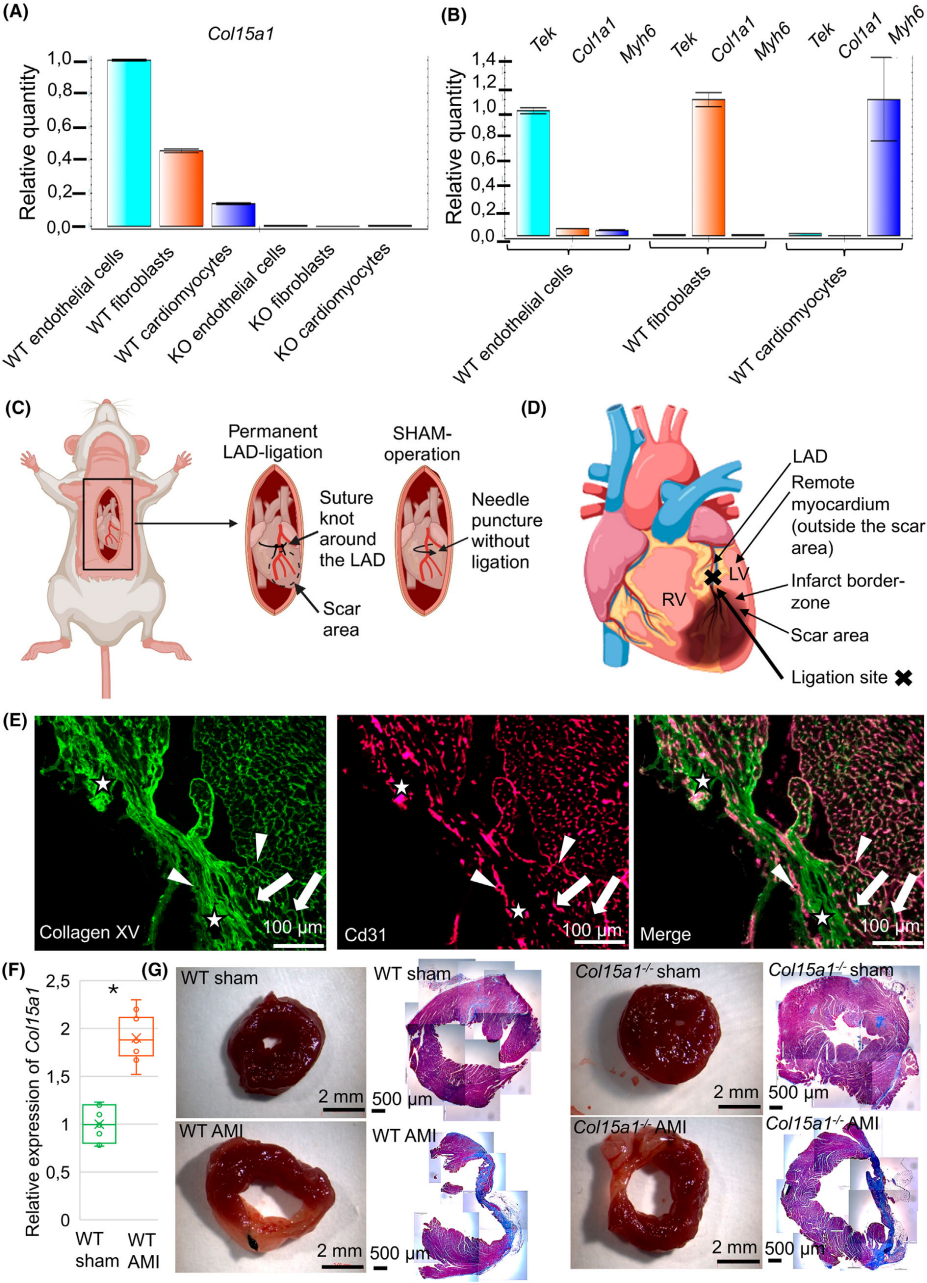
Expression and localisation of collagen XV in fractionated cardiac cells and in the acute myocardial infarction (AMI) scar, and representation of the infarct model and heart morphology in mice. Quantitative PCR analysis of the expression of *Col1a1*, *Myh6, Tek* (*Tie2*) and *Col15a1* mRNA in fractionated mouse cardiac cells (WT hearts, *n* = 5; *Col15a1* ^*−/−*^ hearts, *n* = 5) showed that (A) the collagen XV was mainly expressed in endothelial cells and fibroblasts, showing a lower relative expression level in cardiomyocytes. Cells isolated from *Col15a1* ^*−/−*^ mice did not express *Col15a1*. (B) The expression levels of markers for endothelial cells, fibroblasts and cardiomyocytes [e.g. *Tek* (*Tie2*), *Col1a1* and *Myh6*, respectively] in different cell types indicated that the isolated cell fractions contained specific cell types (duplicate analyses, *n* = 5 mice/group). (C) Schematic representation of the left anterior descending (LAD) coronary artery ligation to induce *in vivo* ischemia in mice. Sham‐control operation was done without the ligation of LAD. (D) Schematic figure of the heart, showing the different areas analysed: scar, border zone and remote myocardium. Ligation site is marked with a thick black cross. (E) Representative image shows that immunofluorescence ColXV signal in the WT mouse (*n* = 7) scar area after AMI localises to the BM zones of the capillaries (arrowheads), the cardiomyocytes (arrows) and the interstitial matrix (stars). Cd31 was used as a marker for endothelial cells. (F) qPCR analysis for *Col151a* in the scar area (the apex of the heart) 5 weeks after AMI (duplicate analyses, *n* = 6 mice/group). (G) Representative images of the mid‐left ventricles of the sham‐operated (WT hearts, *n* = 5; *Col15a1* ^*−/−*^ hearts, *n* = 6) and AMI mice (*n* = 7 mice/group) and the corresponding Masson trichrome‐stained tissue sections, showing the fibrotic scar area (blue). Error bars represent the standard deviation (SD). Original magnifications: E, 20×; G, (Masson staining) 4×. AMI, acute myocardial infarction; KO, knock out (*Col15a1* ^*−/−*^); LAD, left anterior descending coronary artery; LV, left ventricle; RV, right ventricle; WT, wild‐type; *P < 0.05; (Student's *t*‐test). Schematic figures were created in BioRender; Karppinen, S‐M (2025).

To assess the expression of ColXV in different cardiac cells (i.e. cardiomyocytes, fibroblasts and endothelial cells), the cells were fractionated from WT and *Col15a1* ^*−/−*^ mouse hearts and analysed with qPCR. *Col15a1* RNA was mainly expressed by endothelial cells and fibroblasts and was relatively lower in cardiomyocytes (Fig. 2A). These data are in line with the *Col15a1* expression data available in the public Human Protein Atlas database (https://www.proteinatlas.org/ENSG00000204291‐COL15A1/celltype). Previously, ColXV was reported to be primarily produced by endothelial cells and cells originating from the mesenchyme [7]. The qPCR results also showed that cells isolated from *Col15a1* ^*−/−*^ mice did not express *Col15a1* (Fig. 2A) and that the isolated cell fractions were not contaminated by other cell types (Fig. 2B).

### The mouse infarction model showed high ColXV expression in the scar area

To experimentally address the role of ColXV in cardiac infarction, an LAD ligation model was utilised to study the effects of AMI in mice (Fig. 2C). Three areas of the infarcted LV were analysed for different features (Fig. 2D). According to immunofluorescence staining, the AMI scar was highly positive for ColXV (Fig. 2E). Additionally, qPCR showed significantly increased (almost twofold) *Col15a1* expression in the infarcted area in WT AMI animals compared with the sham‐operated group (Fig. 2F) (*P* < 0.05). The signal localisation in the infarcted myocardium was similar to that in human samples, supporting the observation of an increased ColXV signal in fibrotic areas after AMI. Tissue preparations of the mid‐left ventricles were imaged 5 weeks after AMI to visualise the scar area (Fig. 2G).

### Altered cardiomyocyte characteristics and increased heart stiffness in mice lacking ColXV

**Fig. 3:**
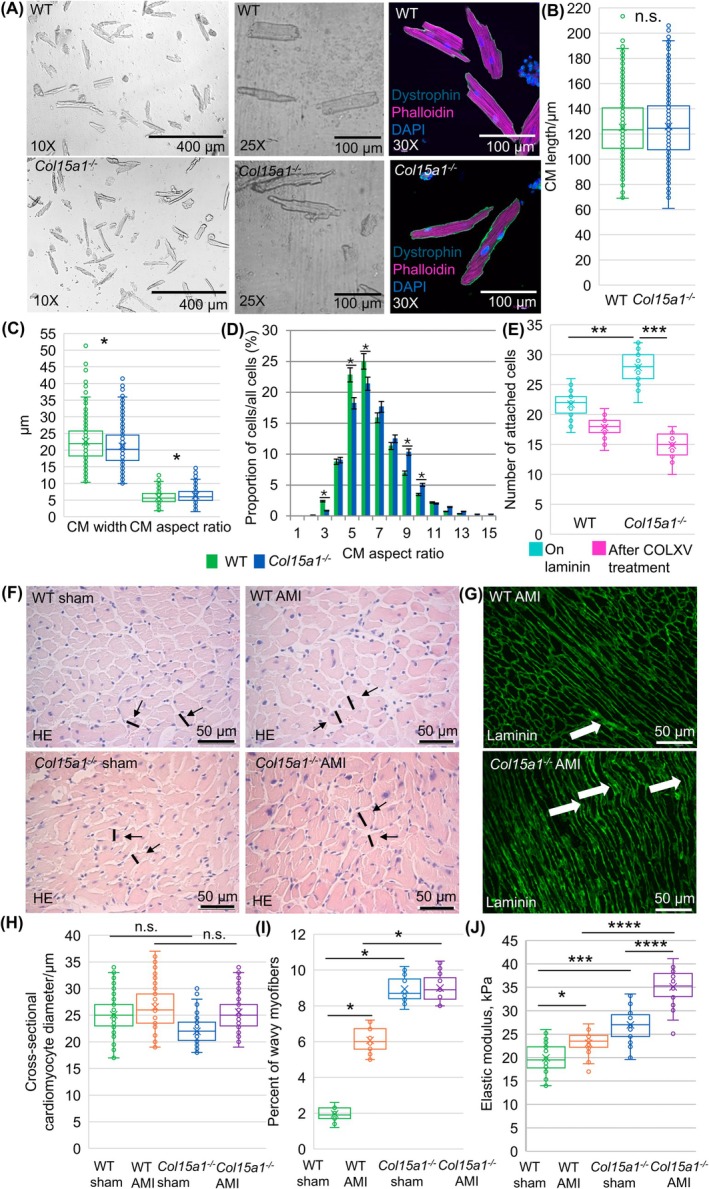
Analyses of cardiomyocytes and heart elasticity in wild‐type and *Col15a1* ^*−/−*^ mice. (A) Representative phase‐contrast images and dystrophin/phalloidin staining of adult mouse primary cardiomyocytes cultured in laminin‐coated wells for 6 h. (B) The lengths and (C) widths of the cardiomyocytes (CM) were measured from the 10× microscopic fields (3 mice/genotype, 5 fields/mouse in (A–D) and (C) the length‐to‐width aspect ratio was counted. (D) The histogram shows the relative frequency distribution of the aspect ratios of the WT and *Col15a1* ^*−/−*^ cardiomyocytes. (E) The numbers of attached cardiomyocytes were counted from the 10× microscopic fields (four mice/genotype, five fields/mouse) of laminin‐coated wells and following treatment with recombinant collagen XV (COLXV). (F) Cross‐sectional HE sections and (G) longitudinal laminin‐stained sections were used to define cardiomyocyte characteristics. (H) The mean diameter (black line) of the cardiomyocytes (black arrows) were measured in the *Col15a1* ^*−/−*^ and WT hearts. (I) Abnormally shaped wavy myofibres (white arrows in B) as percentages in the microscopic fields were analysed after AMI. Ten separate 40× microscopic fields from three mice per genotype were analysed in (F–I). (J) The elastic modulus was calculated for the sham‐operated and AMI hearts (*n* = 4 mice per group, WT hearts, *n* = 3; *Col15a1* ^*−/−*^ hearts, *n* = 4 samples/group, and three measurements of each sample were performed). Error bars represent SD. Original magnifications: (A), 10×, 20× and 60×; (F, G) 40×. *P < 0.05; **P < 0.01; ***P < 0.001; ****P < 0.0001 (nested *t*‐test, nested one‐way ANOVA followed by Tukey's HSD (alpha = 0.05), or chi‐squared and Fisher's exact test); AMI, acute myocardial infarction; CM, cardiomyocyte; COLXV, collagen XV; HE, haematoxylin–eosin; n.s., not significant; WT, wild‐type.

Quantification of the cellular dimensions of primary cardiomyocytes (Fig. 3A) indicated that the absence of ColXV led to the thinning (22.51 μm in WT and 21.29 μm in *Col15a1* ^*−/−*^; *P* < 0.05) and elongation (125.27 μm in WT and 125.99 μm in *Col15a1* ^*−/−*^; n.s.) of the cells (Fig. 3B,C). This caused a significantly increased length‐to‐width aspect ratio (mean value of 5.92 ± 1.83 μm in WT and 6.33 ± 2.01 μm in *Col15a1* ^*−/−*^; *P* < 0.05; Fig. 3C) in *Col15a1* ^*−/−*^ cardiomyocytes compared with WT cardiomyocytes. The histogram of the relative frequency distribution of the cardiomyocyte aspect ratios showed that the relative number of *Col15a1* ^*−/−*^ cells was significantly higher in the groups of cells with aspect ratios of 9 and 10 μm while WT cells dominated in the groups with lower aspect ratios (*P* < 0.05; for WT group median = 6 μm and mode = 137 μm and for *Col15a1* ^*−/−*^ group median = 7 μm and mode = 149 μm) (Fig. 3D). In addition, a significantly higher number of attached cells were observed in the *Col15a1* ^*−/−*^ cardiomyocyte cultures compared with WT cultures (an average of 22 cells in WT and 28 in *Col15a1* ^*−/−*^ per 10x microscopic field; *P* < 0.01), suggesting a possible role of ColXV in affecting the adhesive properties of cardiomyocytes (Fig. 3E). These results were supported by the observation that adding recombinant ColXV in the culture plates decreased the number of attached cardiomyocytes of both genotypes, with the effect being stronger and significant (*P* < 0.001) in knockout cells (an average of 18 cells in WT and 15 in *Col15a1* ^*−/−*^ per 10× microscopic field; Fig. 3E).

Cardiomyocyte characteristics were also analysed from cross‐sectional (Fig. 3F) and longitudinal (Fig. 3G) HE‐stained tissue samples. The cross‐sectional diameter of the *Col15a1* ^*−/−*^ cardiomyocytes was slightly, but not significantly, smaller than the WT samples (25.16 and 26.46 μm in AMI mice, and 21.72 and 25.32 μm in the sham‐operated controls, respectively; Fig. 3H). Additionally, cardiomyocytes that lost their typical rectangle‐like shape and had a wavy organisation were more common in *Col15a1* ^*−/−*^ mice than in WT mice (9% and 6% in the AMI mice, and 9% and 2% in the sham‐operated controls, respectively; *P* < 0.05; Fig. 3I).

Cardiac elasticity was measured using a microprobe intender device, and Young's modulus was calculated. In the sham‐operated *Col15a1* ^*−/−*^ hearts, the cardiac elastic modulus was significantly higher, indicating stiffer tissue compared with the WT hearts (27.0 and 20.1 kPa, respectively; *P* < 0.001) (Fig. 3J). Moreover, infarction promoted more strongly heart stiffness in *Col15a1* ^*−/−*^ mice than in the WT littermates when compared with the baseline values (23.2 kPa in WT AMI; *P* < 0.05 and 35.1 kPa in *Col15a1* ^*−/−*^ AMI; *P* < 0.0001) (Fig. 3J).

### ColXV knockout mice had more fibrosis and proliferative cells in the remote myocardium but no changes in apoptotic, oedemic, necrotic or inflammatory profiles compared with WT mice

**Fig. 4:**
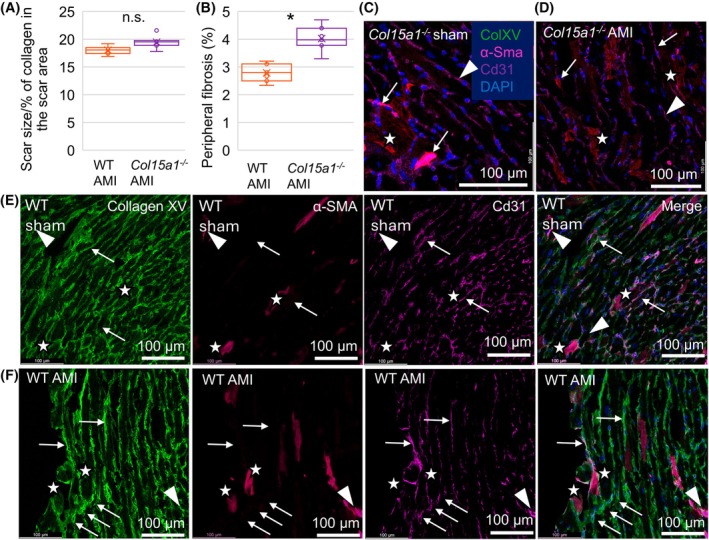
Quantification of scar size and peripheral fibrosis, and co‐localisation of collagen XV with α‐SMA and Cd31 in WT and *Col15a1* ^*−/−*^ hearts. The amount of fibrosis was quantified from the Masson trichrome‐stained sections (A) in the scar area and (B) in the remote myocardium after AMI in the WT and *Col15a1* ^*−/−*^ mice (*n* = 7/genotype). Representative triple‐label IF staining of 20× microscopic fields (3 mice/genotype, 3 fields/mouse) with antibodies against Cd31 (magenta), collagen XV (green) and α‐SMA (purple) shows that (C) α‐SMA and Cd31 co‐localise in *Col15a1* ^*−/−*^ (C) sham and (D) AMI hearts in larger vascular structures (arrows) but not in the spindle shaped cells (stars) or in smaller vascular structures (arrow heads). Collagen XV shows negative staining in *Col15a1* ^*−/−*^ hearts. (E–F) Collagen XV co‐localises with some α‐SMA ‐positive cells in the WT scar area (stars), and additionally with CD31 in vascular structures (arrows) and with both α‐SMA and Cd31 in larger vascular structures (arrow head). DAPI (blue) was used for nuclear staining. Error bars represent SD. Original magnifications 20×. *P < 0.05 (Student's *t*‐test); α‐Sma, alpha‐smooth muscle Actin; AMI, acute myocardial infarction; ColXV, collagen XV; ns., not significant; WT, wild‐type.

**Fig. 5:**
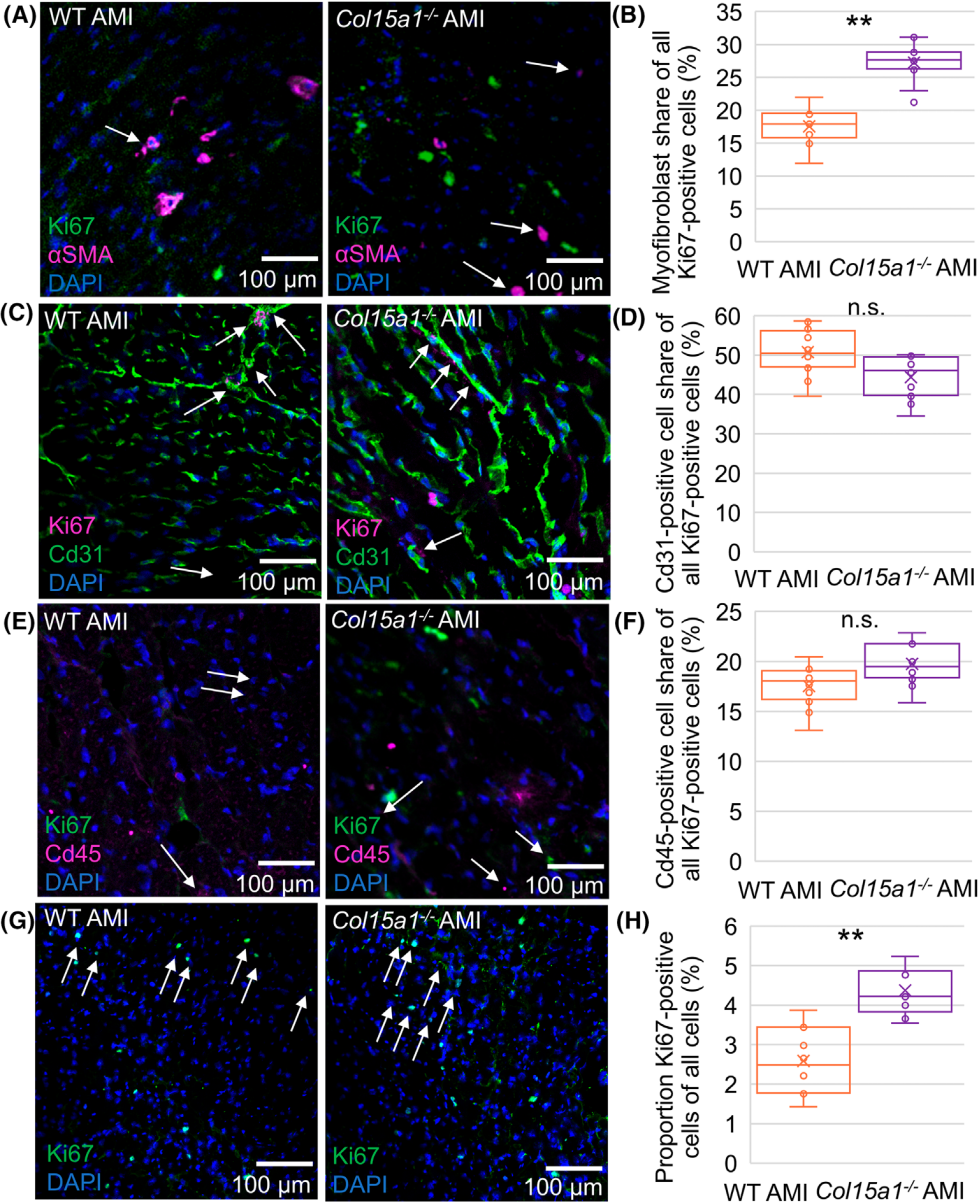
Proliferative cell counts from cryosections of WT and *Col15a1* ^*−/−*^ mice. (A) Proliferating myofibroblasts (arrows) shown in the 20× microscopic fields of the α‐SMA/Ki67‐double‐stained cryosections. (B) The proportions of proliferating myofibroblasts of all proliferating cells were counted from α‐SMA/Ki67 double‐stained cryosections, and proliferating (C–D) immune cells and (E–F) endothelial cells (arrows) of all proliferating cells from CD45/Ki67‐ and CD31/Ki67‐double‐stained cryosections, respectively. (G–H) The proportions of proliferating cells (Ki‐67 positive cells, arrows) of all cells (DAPI) were counted from DAPI/Ki67‐stained cryosections from the remote myocardium of the infarcted WT and *Col15a1* ^*−/−*^ hearts. Ki67 was used as a marker for proliferating cells. 20× microscopic fields (four mice/genotype, three fields/mouse) were analysed at each point. Error bars represent SD. **P < 0.01 (nested *t*‐test); α‐SMA, alpha‐smooth muscle Actin; AMI, acute myocardial infarction; ColXV, collagen XV; ns., not significant; WT, wild‐type.

**Fig. 6:**
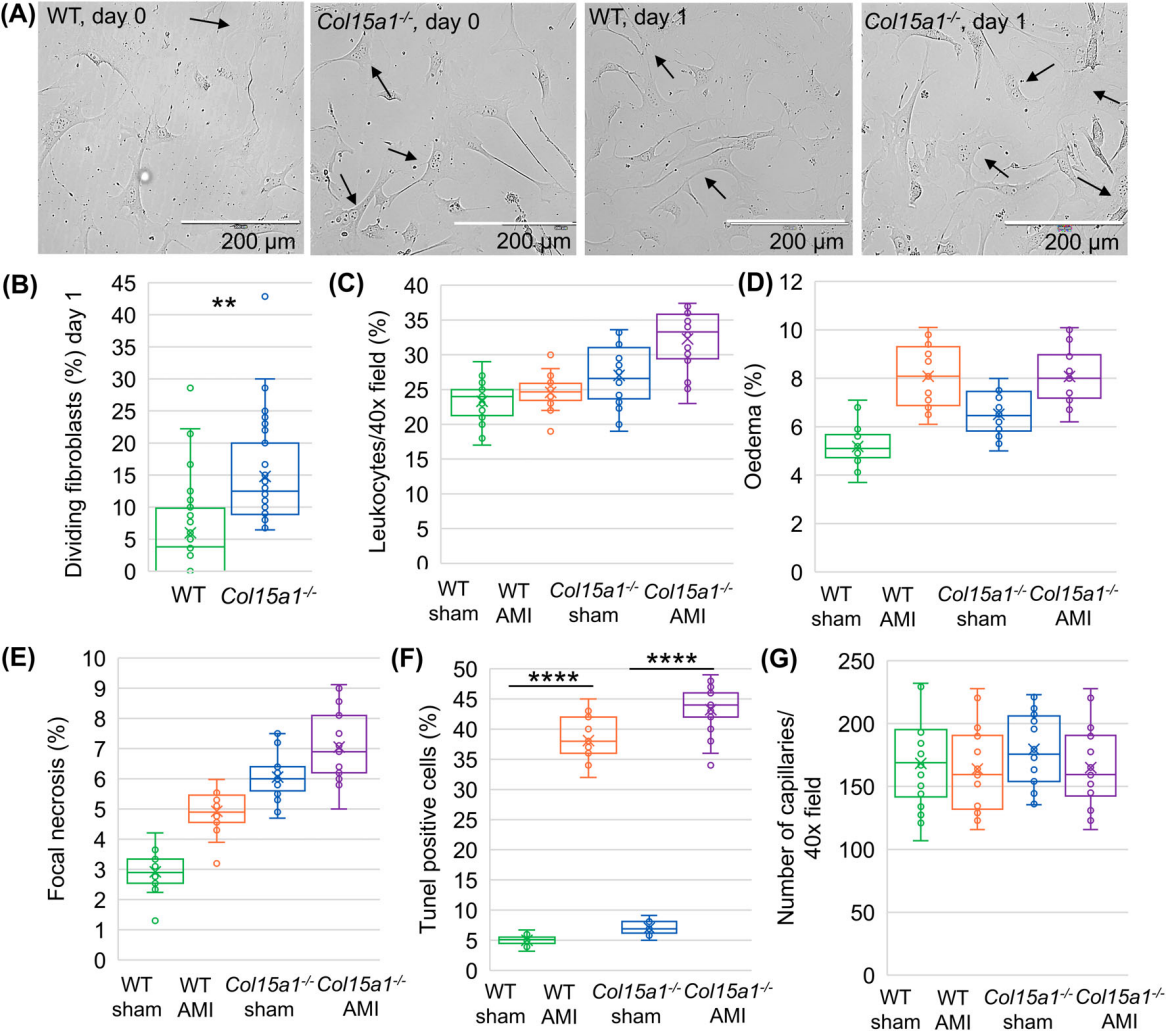
Isolated, cultured fibroblasts and cell counts after AMI in WT and *Col15a1* ^−/−^ hearts. (A) Adult mouse primary fibroblasts were photographed under phase‐contrast microscopy after 6 h (Day 0) and 24 h (Day 1) in representative culture plates. Dividing fibroblasts are indicated by arrows. (B) The fibroblast numbers were counted 24 h after culturing from five 20× microscopic fields from four mice per genotype. Statistical differences were not observed in the quantities of (C) leukocytes, (D) oedema, (E) focal necrosis and (F) apoptotic cells, nor in (G) the average capillary counts between genotypes. Ten separate 40× microscopic fields from three mice per genotype were analysed for C–G. Error bars represent SD. Original magnifications 20×. **P < 0.01; ****P < 0.0001 [nested *t*‐test or nested one‐way ANOVA followed by Tukey's HSD (alpha = 0.05)]. AMI, acute myocardial infarction; WT, wild‐type.

The size of the infarction scar was evaluated by distinguishing the connective tissue in the fibrotic area from the nonfibrotic cardiac muscle in Masson trichrome‐stained cross‐sections obtained from the middle layer of the LV (Fig. 2G). Scar size did not change between the genotypes (the amount of fibrotic area was 17.9% in WT and 20.3% in *Col15a1* ^*−/−*^ mice; n.s.), whereas the fibrosis outside the scar area increased significantly in *Col15a1* ^*−/−*^ mice compared with WT mice (2.9% in WT and 4.1% in *Col15a1* ^*−/−*^ mice; *P* < 0.05) (Fig. 4A,B). In the sham‐operated mice, the amount of fibrotic area was 1.8% in WT and 2.8% in *Col15a1* ^*−/−*^ hearts (n.s.) (Fig. 2G). Based on the α‐SMA and ColXV immunosignals, ColXV co‐localised with some myofibroblasts in the fibrotic area (Fig. 4C–F). Furthermore, the number of myofibroblasts (an average of 11.7 cells in WT and 18.4 cells in *Col15a1* ^*−/−*^ per 10x microscopic field; *P* < 0.001) and proliferating myofibroblasts counted from Ki‐67/α‐SMA double‐stained AMI tissue sections was higher in *Col15a1* ^*−/−*^ than in WT cryosections (17.6% of all Ki‐67‐positive cells in WT and 27.3% in *Col15a1* ^*−/−*^; *P* < 0.01) (Fig. 5A,B). The relative amounts of proliferating endothelial (Cd31/Ki‐67 positive) and inflammatory (Cd45/Ki‐67 positive) cells were not statistically significant between the genotypes (Fig. 5C–F). The total quantity of Ki‐67‐positive cells among all cells was higher in *Col15a1* ^*−/−*^ mice than in WT animals in the remote myocardium (2.6% in WT and 4.3% in *Col15a1* ^*−/−*^; *P* < 0.01) (Fig. 5G,H). Morphological differences between the genotypes were not seen in isolated primary fibroblasts; however, we observed significantly more dividing fibroblasts in the *Col15a1* ^*−/−*^ cultures 24 h after plating (5.9% in WT and 14.7% in *Col15a1* ^*−/−*^; *P* < 0.01) (Fig. 6A,B).

According to the HE section analysis, the average leukocyte counts and relative amounts of oedemic and necrotic areas in the AMI scars were slightly elevated in both genotypes compared with sham‐operated animals during the scar maturation and myocardial remodelling phases. No difference between the genotypes was observed (Fig. 6C–E). As expected, the TUNEL‐stained sections showed increased apoptotic cell counts in the ischemic samples compared with the sham‐operated samples, although the amount was similar between the genotypes (Fig. 6F). Furthermore, there was no change in the number of myocardial capillaries assessed in the CD31‐stained sections (Fig. 6G).

### The composition and organisation of the scar border zone and scar area were compromised in the *Col15a1* ^*−/−*^ heart

**Fig. 7:**
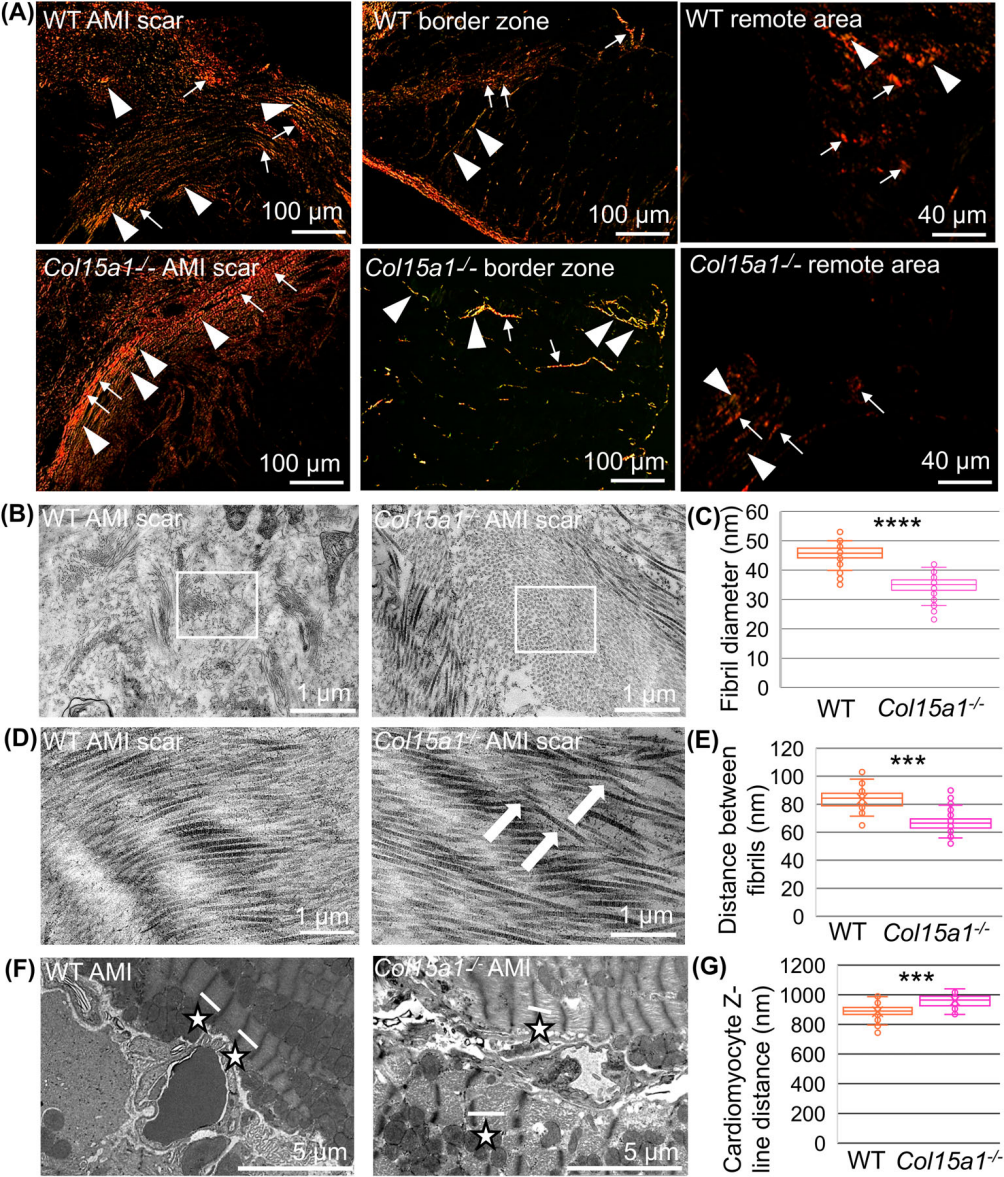
Histological and ultrastructural analyses of collagen fibres and sarcomere structure. (A) Picrosirius red was used to stain the thin (green, arrowheads) and thick (red, arrows) collagen fibres in the scar, scar border zone and remote myocardium. (B) Representative transmission electron microscopy (TEM) images show different proportions of poorly visible areas of cross‐sectional collagen fibres (squared area in the WT sample) and areas where the fibres were readily visualised (squared area in the *Col15a1* ^*−/−*^ sample) between the genotypes (*P* < 0.0001, nested *t*‐test). (C) The diameters of the fibrils were measured from the TEM images. (D) Longitudinal sections of collagen fibres revealed disorganised areas of the collagenous matrix in *Col15a1* ^*−/−*^ scars (arrows). (E) The distances between the fibrils were measured from the TEM images, and (F–G) the distance between the Z‐lines (indicated by stars and white lines) was analysed. Ten separate microscopic fields for light microscopy (A) and five fields for TEM (B–G) from three mice per genotype were analysed. Original magnifications: A 20×; B and D 11000×; F 7400×. Error bars represent SD. ****P < 0.0001 (nested *t*‐test); AMI, acute myocardial infarction; WT, wild‐type.

Picrosirius red staining under polarised light microscopy was performed to investigate the collagen fibres. The ratio of thick (red, mature) to thin (green, immature) collagen fibres was significantly lower in the fibrotic tissue within the scar border zone for the *Col15a1* ^*−/−*^ mice (1.41 in WT and 1.13 in *Col15a1* ^*−/−*^; *P* < 0.05) (Fig. 7A), implying a significantly increased proportion of immature fibres relative to mature fibres. There was no difference between the genotypes concerning the ratio in the central scar area (2.43 in WT and 2.37 in *Col15a1* ^*−/−*^; n.s.) or in the remote area (1.49 in WT and 1.23 in *Col15a1* ^*−/−*^; n.s.) (Fig. 7A).

Transmission electron microscopy (TEM) was used to study scar ultrastructure, revealing striking differences between the genotypes. Cross‐sectional collagen fibres appeared as poorly identifiable patches in the WT samples, whereas they were clearly visible and abundant in the *Col15a1* ^*−/−*^ scars (Fig. 7B). In more detail, the fibres were clearly visible in 31% of the analysed areas of the WT samples compared with 75% of the *Col15a1* ^*−/−*^ samples (*P* < 0.0001; nested *t*‐test, five fields from three mice per genotype were analysed). The different appearances between the genotypes suggested changes in the amorphous material and microfibrillar components, probably in the elastic fibre microfibrils [16, 17]. The mean diameter of the cross‐sectional collagen fibrils in the *Col15a1* ^*−/−*^ scars was significantly lower than the WT scars (35.0 and 45.5 nm, respectively; *P* < 0.0001) (Fig. 7C). Fibres in the longitudinal sections appeared well‐organised in the WT scars, whereas they frequently crossed each other in the *Col15a1* ^*−/−*^ scars, indicating disturbed organisation (Fig. 7D). Additionally, the mean distance between individual fibrils was smaller in the *Col15a1* ^*−/−*^ than in the WT scar (66.8 and 83.9 nm, respectively; *P* < 0.001) (Fig. 7E).

The sarcomere structure and the distance between the Z‐discs were analysed by TEM to study the ultrastructure and myofibrillar architecture of cardiomyocytes. The mean distance between Z‐lines in the WT mice was shorter than in the *Col15a1* ^*−/−*^ cardiomyocytes (886 and 958 nm, respectively; *P* < 0.001) (Fig. 7F,G). Furthermore, the sarcomere structure was more often compromised in the *Col15a1* ^*−/−*^ mice than in the WT mice.

### The amount of fibrillin‐1 was reduced in the *Col15a1* ^*−/−*^ scars

**Fig. 8:**
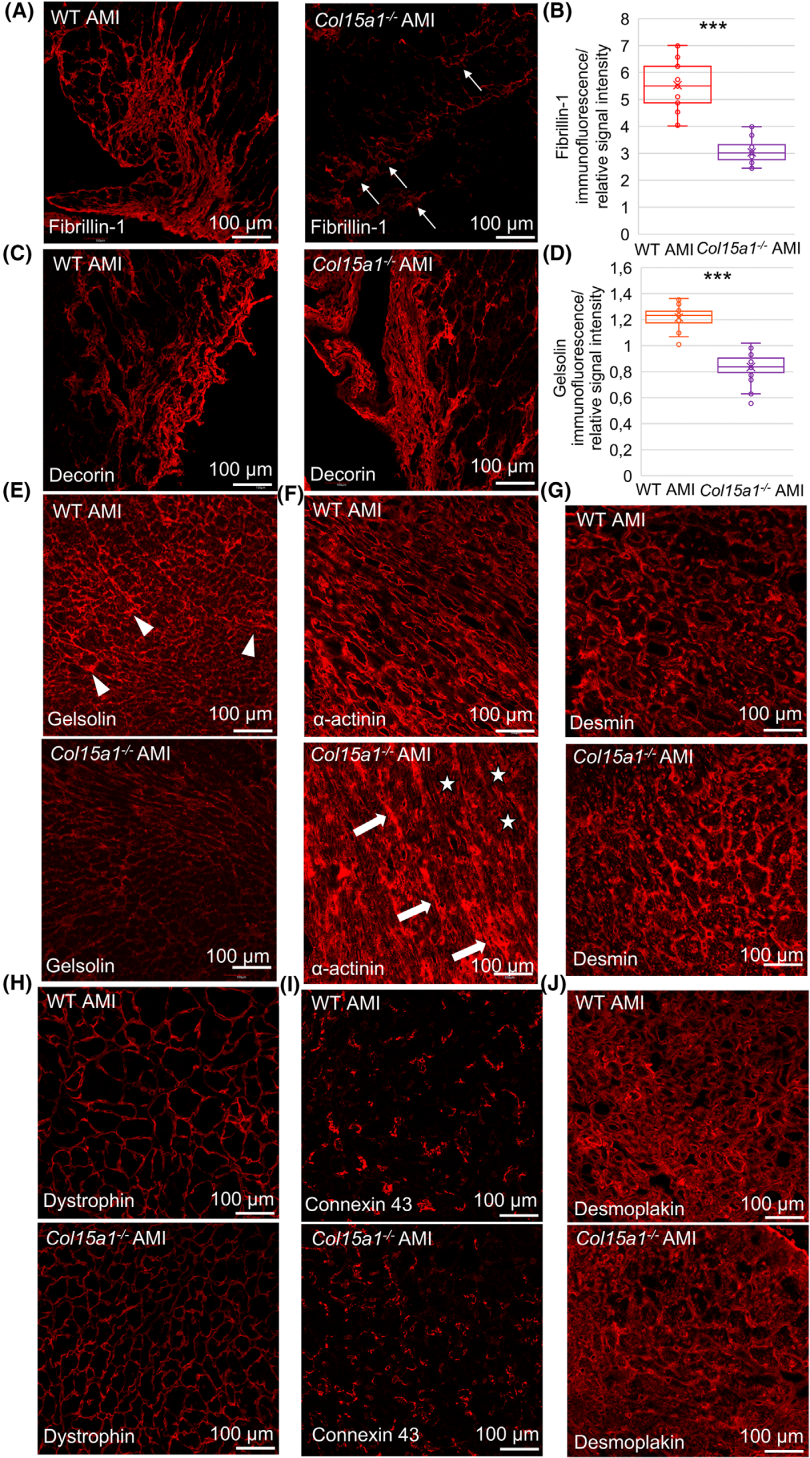
Immunofluorescence analyses after AMI in mouse hearts. (A) The fibrillin‐1 immunosignal was highly reduced and focally aggregated (arrows) in the *Col15a1* ^*−/−*^ infarction scars compared with the stronger and fibrillar‐like signal in the WT scars. (B) Quantification of Fibrillin‐1 immunofluorescence signal. (C) The signal for decorin was slightly but not significantly decreased in the *Col15a1* ^*−/−*^ scars. (D–E) The signal for gelsolin was highly downregulated in the *Col15a1* ^*−/−*^ remote area. Perivascular areas often had high gelsolin signals (arrowheads). (F) The alpha‐actinin signal was unevenly distributed in the *Col15a1* ^*−/−*^ remote area, showing both low‐ (stars) and high‐ (arrows) intensity areas. Equal signals for (G) desmin, (H) dystrophin, (I) connexin 43 and (J) desmoplakin were observed between WT and *Col15a1* ^*−/−*^ remote area. Ten separate 20× microscopic fields from three mice per genotype were analysed for each staining. Error bars represent SD. ***P < 0.001 (nested *t*‐test); AMI, acute myocardial infarction; WT, wild‐type.

Due to evidence of altered microfibrillar components in the *Col15a1* ^*−/−*^ scar in the ultrastructural analysis, immunofluorescence signal for fibrillin‐1 and decorin was quantified. A significantly reduced (*P* < 0.001) mean signal intensity of fibrillin‐1 in the *Col15a1* ^*−/−*^ scars was observed compared with the WT scars (Fig. 8A,B). The immunofluorescence signal of decorin, a proteoglycan associated with collagen fibrils and fibrillin‐1, was slightly but not significantly reduced in the *Col15a1* ^*−/−*^ scar area (Fig. 8C).

### Analysis of cytoskeletal proteins in postinfarcted left ventricles revealed differences in the expression of gelsolin, α‐actinin and desmin between the genotypes

Due to the sarcomeric imbalance in *Col15a1* ^*−/−*^ LVs, immunofluorescence staining with markers for several cytoskeletal proteins (e.g. the Z‐disc marker α‐actinin, actin‐binding protein gelsolin, intermediate filament desmin, desmosome junction protein desmoplakin, gap‐junction protein connexin 43 and anchor protein dystrophin) was performed.

In the heart, the mechanosensing protein gelsolin is typically located both in the extracellular and intracellular spaces [18]. Immunofluorescence analysis of the AMI samples showed significantly reduced extracellular gelsolin expression in the *Col15a1* ^*−/−*^ LVs compared with the WT hearts (*P* < 0.001) (Fig. 8D,E). Quantitative PCR supported the immunofluorescence analysis, indicating downregulated gelsolin expression in the *Col15a1* ^*−/−*^ AMI mice (FC = −2.3; *P* < 0.001, Table 1). The immunofluorescence signal of α‐actinin was evenly distributed in the WT samples after AMI, whereas in the *Col15a1* ^*−/−*^ samples, the signal was often unevenly distributed and had areas of both elevated and decreased intensity (Fig. 8F). In the *Col15a1* ^*−/−*^ mice, the desmin immunofluorescence signal increased slightly but not statistically after AMI compared with the WT mice (Fig. 8G). Dystrophin, desmoplakin and connexin 43 signals were also equal between the genotypes (Fig. 8H–J).

**Table 1:** Fold changes in the qPCR analysis between the wild‐type (WT) and *Col15a1* ^*−/−*^ remote left ventricles after acute myocardial infarction (AMI) (*n* = 5 per genotype).

Gene	Protein	Sample	Fold change vs. wild‐type	*P*‐value
*Col1a1*	Collagen type I, alpha 1	AMI	3.0	0.0002***
*Col3a1*	Collagen type III, alpha 1	AMI	2.9	0.0004***
*Dpt*	Dermatopontin	AMI	−1.5	0.01
*Gsn*	Gelsolin	AMI	−2.3	0.0003***
*Loxl1*	Lysyl oxidase like 1	AMI	2.3	0.001
*Loxl2*	Lysyl oxidase like 2	AMI	1.8	0.003
*Loxl3*	Lysyl oxidase like 3	AMI	3.8	0.000003***
*Myh7*	Myosin heavy chain 7	AMI	5.2	0.00005***
*Nppa*	Atrial natriuretic peptide	AMI	5.5	0.0000000009***
*Nppb*	Brain natriuretic peptide	AMI	1.4	0.004
*Tgfb1*	Transforming growth factor beta 1	AMI	1.5	0.02
*Ttn*	Titin	AMI	5.8	0.0002***
*Vcl*	Vinculin	AMI	−2.1	0.004

*** Significant; *P*‐value cut‐off, *P* < 0.001 (Student's *t*‐test)

### Lack of collagen XV resulted in a more substantial myocardial remodelling process and a worse outcome after cardiac ischemia

In a previous echocardiographic study of 14‐month‐old mice, female *Col15a1* ^*−/−*^ mice had significantly thinner posterior ventricular walls (LVPW), thinner interventricular septa (IVS) and diminished left ventricular mass (LV mass) compared with WT mice [15]. In this study, our aim was to study the cardiac parameters in male mice and compare those with previous data of female mice which had a phenotype resembling DCM. The preoperative echocardiographic measurements revealed no differences in 18–24‐month‐old male mice (Table 2) between the genotypes. Thus, unexpectedly, heart function in the *Col15a1* ^*−/−*^ mice might have gender‐dependent effects which were not recognised previously. Here we aimed to study the effect of AMI in male mice to see whether the lack of collagen XV can lead to worse outcomes even without any preobserved cardiac changes.

**Table 2:** Numerical data of echocardiographic measurements and heart and body weights for wild‐type (WT) and *Col15a1* ^*−/−*^ mice before subjected to acute myocardial infarction (AMI), as well as 1 and 5 weeks after AMI. d, diastolic; EF, ejection fraction; ENDOarea d/S, left ventricular end‐diastolic/systolic area; FAC, fractional area change; FS, fractional shortening; IVRT, isovolumetric relaxation time; IVS, interventricular septum; LV, left ventricle; LVID d, left ventricle end‐diastolic diameter; LVPW d, left ventricular posterior wall thickness in diastole; MV Decel, mitral valve deceleration time; s, systolic.

	Preoperative		1 week after AMI				5 weeks after AMI			
	Wild‐type	*Col15a1* ^*−/−*^	Wild‐type sham	Wild‐type AMI^a^	*Col15a1* ^*−/−*^ sham^b^	*Col15a1* ^*−/−*^ AMI^a^, ^b^	Wild‐type sham	Wild‐type AMI^a^	*Col15a1* ^*−/−*^ sham^b^	*Col15a1* ^*−/−* AMI^ ^a^, ^b^
ENDOarea d (mm^2^)	15.29 ± 3.50	12.59 ± 2.42	12.01 ± 1.68	14.92 ± 2.86	12.10 ± 1.33	20.24 ± 2.73**^,^***	13.15 ± 1.29	19.43 ± 4.80*	12.59 ± 2.01	23.45 ± 5.87^‐,^**
ENDOarea s (mm^2^)	9.59 ± 3.38	6.90 ± 1.95	6.04 ± 2.40	10.53 ± 4.12	6.62 ± 1.78	16.54 ± 3.74*^,^***	7.72 ± 1.22	15.33 ± 5.14*	6.96 ± 0.87	19.30 ± 5.70^‐,^***
LVID, d (mm)	4.60 ± 0.38	4.30 ± 0.30	4.22 ± 0.36	4.70 ± 0.41	4.17 ± 0.25	5.21 ± 0.41*^,^***	4.39 ± 0.24	5.03 ± 0.25**	4.19 ± 0.52	5.38 ± 0.78^‐,^**
LVID, s (mm)	3.43 ± 0.35	3.10 ± 0.37	2.90 ± 0.54	4.04 ± 0.57**	2.99 ± 0.30	4.67 ± 0.48*^,^***	3.34 ± 0.27	4.32 ± 0.31***	3.06 ± 0.43	4.83 ± 0.77^‐,^***
LV volume d (μL)	98.04 ± 16.4	83.49 ± 13.8	80.22 ± 16.0	103.52 ± 20.7	77.68 ± 11.0	130.84 ± 23.4*^,^***	87.63 ± 10.84	120.30 ± 13.7**	79.44 ± 22.21	143.68 ± 46.6^‐,^*
LV volume s (μL)	49.07 ± 10.8	38.64 ± 11.6	33.53 ± 14.8	73.67 ± 24.7*	35.05 ± 8.61	102.22 ± 24.3*^,^***	45.86 ± 8.48	84.35 ± 14.1***	37.73 ± 12.21	112.56 ± 40.8^‐,^**
IVS, d (mm)	0.67 ± 0.10	0.76 ± 0.15	1.00 ± 0.12	0.78 ± 0.22	0.74 ± 0.16*	0.77 ± 0.23	0.87 ± 0.14	0.93 ± 0.12	0.91 ± 0.22	0.73 ± 0.36
IVS, s (mm)	0.91 ± 0.15	1.06 ± 0.23	1.39 ± 0.16	0.91 ± 0.28*	1.00 ± 0.22*	0.95 ± 0.36	1.16 ± 0.18	1.16 ± 0.18	1.22 ± 0.16	0.86 ± 0.43
LVPW, d (mm)	0.72 ± 0.07	0.84 ± 0.11	0.92 ± 0.08	0.66 ± 0.25	0.82 ± 0.09	0.55 ± 0.29	0.90 ± 0.19	0.89 ± 0.14	0.83 ± 0.15	0.75 ± 0.29
LVPW, s (mm)	1.04 ± 0.12	1.19 ± 0.18	1.34 ± 0.10	0.73 ± 0.30**	1.16 ± 0.17	0.61 ± 0.34^‐,^**	1.20 ± 0.23	1.06 ± 0.22	1.10 ± 0.13	0.88 ± 0.38
FS (%)	25.51 ± 3.59	28.03 ± 4.10	31.76 ± 7.54	14.24 ± 6.53**	28.30 ± 8.04	10.41 ± 3.58*^,^***	23.95 ± 3.62	14.43 ± 3.32**	27.01 ± 1.79	10.51 ± 1.62*^,^***
EF (%)	50.20 ± 5.71	54.25 ± 7.07	59.56 ± 11.2	29.87 ± 12.7**	54.31 ± 12.5	22.44 ± 7.31*^,^***	47.82 ± 6.20	30.41 ± 6.37**	53.05 ± 3.12	22.69 ± 3.47*^,^***
IVRT (ms)	14.67 ± 3.47	15.70 ± 2.85	14.72 ± 2.39	14.90 ± 1.39	15.75 ± 4.41	15.41 ± 3.20	15.93 ± 2.21	16.80 ± 4.12	17.84 ± 3.56	22.14 ± 4.84^*,^‐
MV Decel (ms)	16.71 ± 6.31	13.32 ± 4.37	12.77 ± 3.16	9.60 ± 4.36	16.08 ± 5.08	14.21 ± 7.34	13.38 ± 7.05	8.39 ± 4.35	13.04 ± 6.23	6.40 ± 2.80
FAC (%)	38.61 ± 9.59	45.42 ± 9.26	50.94 ± 12.3	30.25 ± 18.5	45.38 ± 13.7	18.95 ± 9.42‐,**	41.56 ± 3.94	22.65 ± 7.68**	44.42 ± 2.98	18.22 ± 7.19‐,***
Body weight (g)	51.92 ± 7.65	41.52 ± 6.03*	43.95 ± 3.96	44.10 ± 4.97	38.32 ± 5.19	37.00 ± 5.23^*,^‐	41.54 ± 3.50	39.93 ± 2.04	41.76 ± 2.70	40.99 ± 2.96
LV weight (mg)	99.45 ± 11.0	106.36 ± 23.2	132.89 ± 18.3	112.12 ± 35.3	99.64 ± 26.2	114.33 ± 31.1	151.60 ± 33.2	207.14 ± 44.1	147.01 ± 24.75	198.33 ± 65.1
LV/Body weight x1000	1.92 ± 0.68	2.49 ± 0.89	3.02 ± 0.68	2.54 ± 0.71	2.60 ± 0.76	3.09 ± 0.67	3.65 ± 0.90	5.19 ± 0.84	3.52 ± 0.68	4.84 ± 1.03

^a^ *P < 0.05; **P < 0.01; ***P < 0.001, sham vs. AMI in each genotype (Student's *t*‐test); (*n* = 5–7 per genotype).

^b^ *P < 0.05; **P < 0.01; vs. corresponding wild‐type group (Student's *t*‐test); (*n* = 5–7 per genotype).

**Fig. 9:**
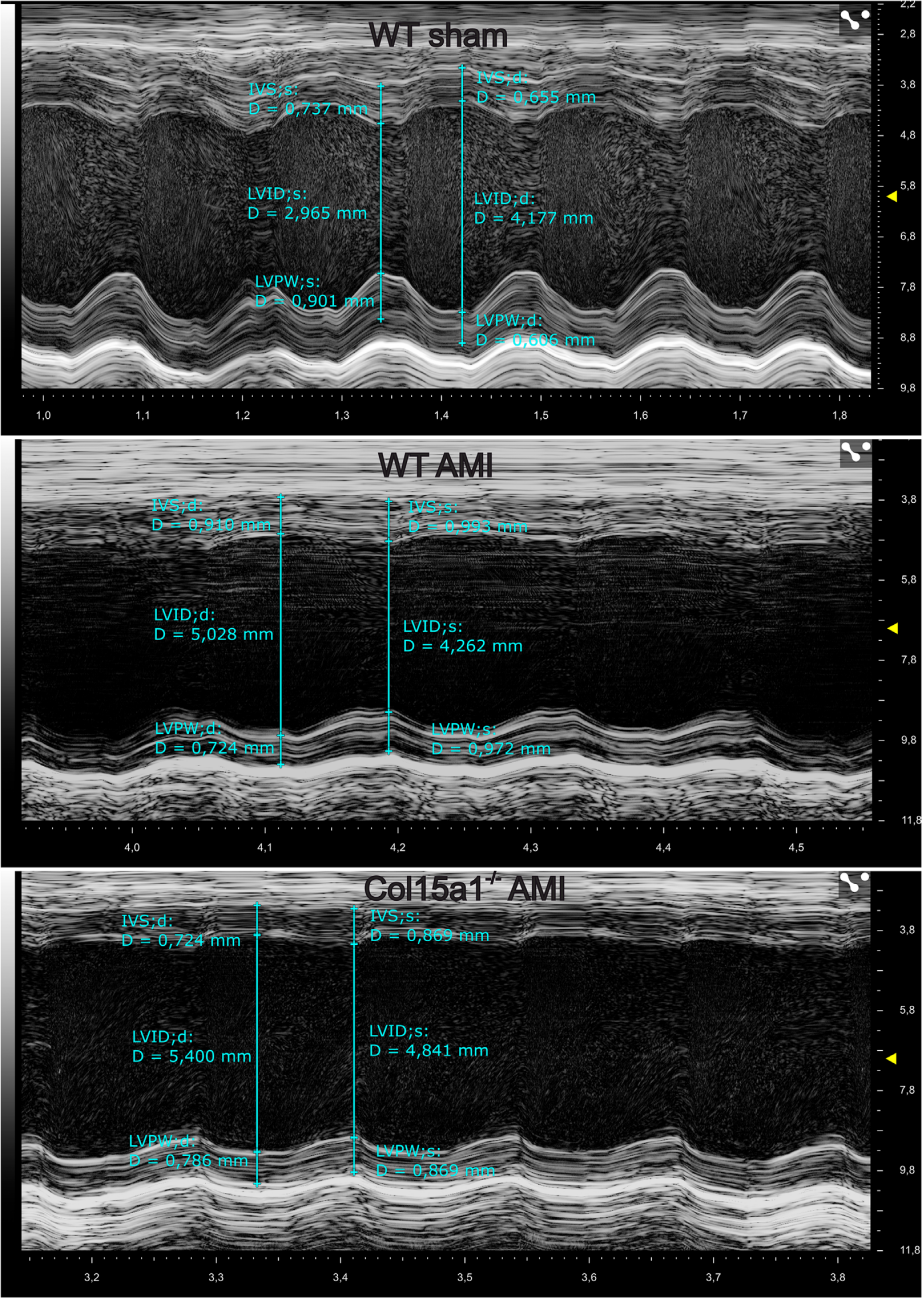
Representative transthoracic M‐mode SAX images showing the measurement of echocardiography parameters. Images of wild‐type sham (noninfarcted) (uppermost figure), wild‐type AMI (middle figure) and *Col15a1* ^*−/−*^ AMI (lowest figure) mice 5 weeks after AMI to show the difference in contractile functions. IVS;d, interventricular septum in diastole; IVS;s, interventricular septum in systole; LVID;d, left ventricular internal diameter in diastole; LVID;s, left ventricular internal diameter in systole; LVPW;d, left ventricular posterior wall thickness in diastole; LVPW;s, left ventricular posterior wall thickness in systole. Vevo Lab 5.8.1 ultrasound analysis software was used to view and measure the acquired data.

Five weeks after being subjected to AMI, the echocardiographic value of EF (cut‐off > 40%) was used to evaluate whether the mice (18–24‐month‐old) had AMI. Representative echocardiography M‐mode images of the short axis (SAX) 5 weeks after AMI are shown in Fig. 9. The difference in survival between the genotypes was not statistically significant. Already 1 week after the operation, significant decreases in the EF and FS were observed in the AMI mice compared with the sham‐operated animals in both genotypes (*P* < 0.01 in the WT mice and *P* < 0.001 in the *Col15a1* ^*−/−*^ mice), indicating defective heart contraction capacity (Table 2). The FAC parameter, also indicating cardiac contractile performance, decreased significantly in the *Col15a1* ^*−/−*^ mice (*P* < 0.01) but not in the WT group. In general, differences in structural parameters between the WT AMI and sham‐operated groups were less prominent and less significant than those in the *Col15a1* ^*−/−*^ AMI mice (Table 2). For example, WT AMI mice showed a modest increase in LV dilatation compared with sham‐operated animals; LV diastolic volume was 80 μL in the WT sham compared with 104 μL in the WT AMI mice (n.s.). On the contrary, the *Col15a1* ^*−/−*^ AMI mice had a sizable increase in LV dilatation compared with the corresponding sham mice; LV diastolic volume was 78 μL in the sham mice and 131 μL in the AMI mice (*P* < 0.001), indicating accelerated post‐MI remodelling.

**Fig. 10:**
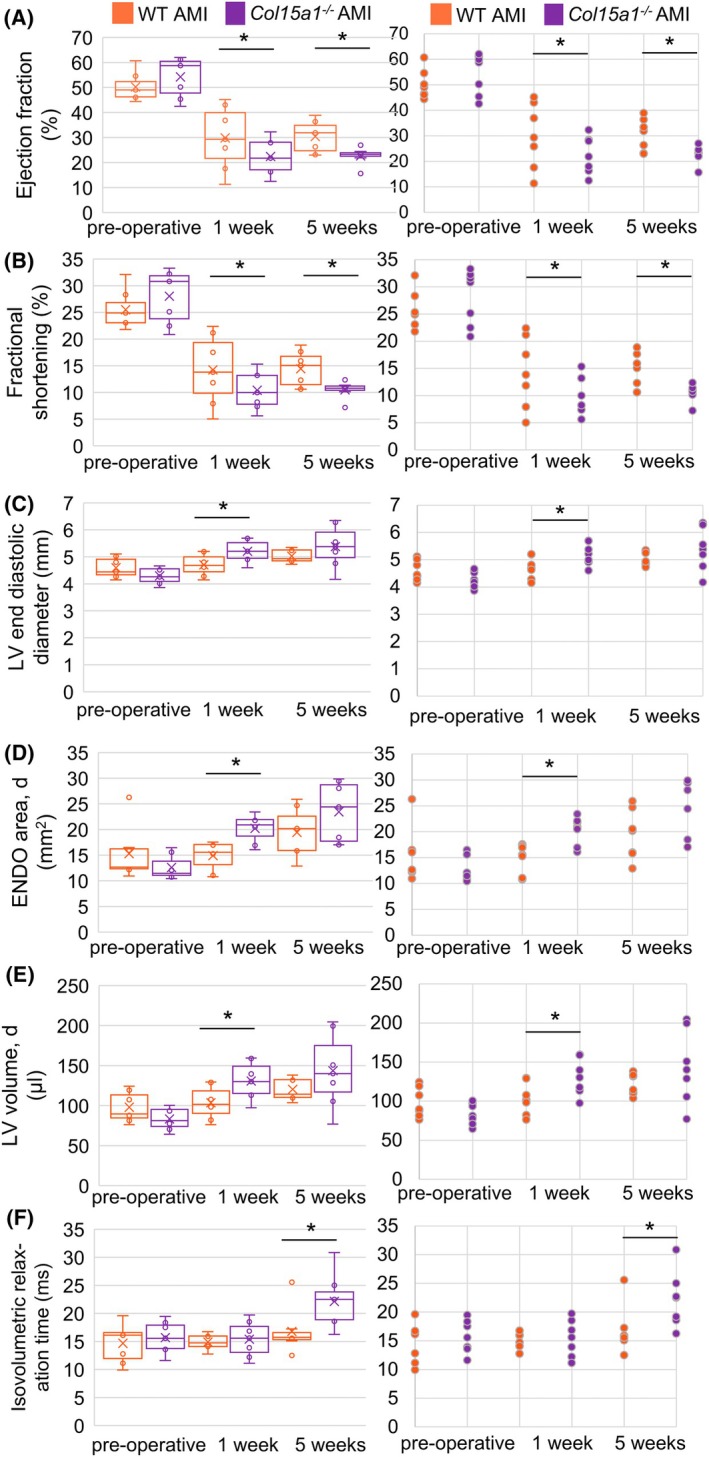
Echocardiography results in mice. The boxplot (left) shows (A) the ejection fraction, (B) fractional shortening, (C) LV end‐diastolic diameter, (D) LV end‐diastolic area (ENDO area; d), (E) left ventricular diastolic volume (LV volume; d) and (F) isovolumic relaxation time at different time points visualised as the median (black line within each box), 25^th^ and 75^th^ percentiles, with whiskers indicating maximal and minimal values (i.e. the variability outside the upper and lower quartiles), and dots denoting outliers. The scatter plot (right) shows individual datapoints. The normal distribution was assessed using the Kolmogorov–Smirnov test. Statistical analysis was performed to compare the two groups using unpaired, two‐tailed Student's *t*‐tests. *n* = 5–7 mice per genotype. *P < 0.05; AMI, acute myocardial infarction; d, diastolic; LV, left ventricle; WT, wild‐type.

At the 1‐ and 5‐week time points after AMI, the *Col15a1* ^*−/−*^ LAD ligated mice had statistically lower (*P* < 0.05) EF (Fig. 10A) and FS (Fig. 10B), both indicators of LV systolic function, than the WT mice. EF was 23% in the *Col15a1* ^*−/−*^ mice and 30% in the WT mice 5 weeks after AMI (Table 2). The LV end‐diastolic diameter (LVID;d) indicated that postinfarction LV remodelling increased significantly in the *Col15a1* ^*−/−*^ mice compared with the WT littermates 1 week after AMI (*P* < 0.05) (Table 2, Fig. 10C). Furthermore, the LV end‐diastolic and end‐systolic areas (ENDOarea) and volumes increased significantly (*P* < 0.05) in the *Col15a1* ^*−/−*^ AMI group at the same time point (Table 2, diastolic values in Fig. 10D,E, respectively). The isovolumetric relaxation time (IVRT), which increases due to impaired LV relaxation, was significantly lengthened in the *Col15a1* ^*−/−*^ mice 5 weeks after AMI (*P* < 0.05) (Fig. 10F).

### Expression of several genes changed in the postinfarcted *Col15a1* ^*−/−*^ left ventricles

Due to increased fibrosis in the remote LV area in the *Col15a1* ^*−/−*^ mice, the expression of transforming growth factor β (*Tgf‐β*), type I and III collagens (*Col1a1* and *Col3a1*) and the lysyl oxidases (*Loxl1, Loxl2* and *Loxl3*) was analysed with quantitative PCR. In addition, the more substantial dysfunction and excessive post‐AMI LV remodelling in the *Col15a1* ^*−/−*^ mice prompted us to study the expression of several other proteins implicated in AMI. The results are summarised in Table 1.

Among the lysyl oxidase‐encoding genes, *Loxl3* was the most upregulated in the *Col15a1* ^*−/−*^ remote LV area, with almost fourfold increased expression compared with respective WT mice (FC = 3.8; *P* < 0.0001) (Table 1). The expression of both *Col1a1* and *Col3a1* increased significantly in the *Col15a1* ^*−/−*^ AMI mice, showing almost threefold change (FC = 2.9; *P* < 0.001) compared with the WT samples. *Tgf‐β* showed slightly but not significantly increased expression in *Col15a1* ^*−/−*^ hearts compared with WT hearts (FC = 1.5; n.s.). Both stress‐induced natriuretic peptides were upregulated after AMI in *Col15a1* ^*−/−*^ LVs compared with WT LVs. The A form (*Nppa*, *Anp*) was significantly upregulated (5.5‐fold; *P* < 0.001), whereas the B form (*Nppb*, *Bnp*) was not (1.4‐fold; n.s.) compared with the WT mice. Among the other studied proteins, titin and myosin heavy chain 7 were highly upregulated in the *Col15a1* ^*−/−*^ mice (FC = 5.8 and FC = 5.2, respectively). Dermatopontin and vinculin encoding genes were slightly but not significantly downregulated (FC = −1.5 and FC = −2.1) (Table 1).

## Discussion

We demonstrated here for the first time that ColXV is highly expressed in infarct scar and has a protective role in the myocardium when cardiac homeostasis is challenged by acute myocardial infarction. We found that ColXV has a wide‐ranging impact after AMI, affecting not only the scar area but also the infarct border zone and the remote myocardium. Our novel findings show that a lack of ColXV leads to maladaptive ECM and LV remodelling characterised by (1) changes in scar structure and composition, suggesting reduced resistance to stretching; (2) an immature and fragile infarct border zone; (3) increased tissue stiffness and fibrosis; (4) aberrant cardiomyocyte characteristics and gene expression; (5) changes in sarcomere structure and cytoskeletal protein composition; and (6) exacerbated diastolic dysfunction, leading to more substantial heart impairment after AMI. These wide effects are most probably accompanied by the previously observed hemodynamic changes and impaired microvascular function in the *Col15a1* ^*−/−*^ mice [15].

ECM plays a fundamental role in myocardial infarction [19]. In an optimal situation, it results in a stable scar that provides structural support to the cardiac muscle. The expression of several ECM proteins increases in the infarcted myocardium [2, 20], and the lack of several of them results in aggravated LV remodelling, indicating their protective postinfarction role. Collagens are the principal scar constituent, and several collagens, including both fibrillar (I, III and V) and nonfibrillar (IV, VI and XVIII) types, have been proven relevant in MI [20]. Recently, urinary proteomics of patients with heart failure showed that high ColXV levels associate with a lower risk of death or heart failure admission [21].

In this study, we observed high ColXV expression in the infarct scar area in humans and mice. Furthermore, the lack of ColXV resulted in an abnormal scar characterised by a disorganised fibrillar collagen pattern and abnormal ECM composition. The scar size and leukocyte infiltration 5 weeks after AMI were equal to WT during the scar maturation phase. The different appearance of collagen fibres in the *Col15a1* ^*−/−*^ scars was hypothesised to be a consequence of reduced amounts of microfibrillar components. Indeed, we observed a significant reduction in the microfibrillar protein fibrillin‐1 and a slight decrease in the amount of the collagen‐associated proteoglycan decorin in the *Col15a1* ^*−/−*^ scars compared with WT scars. Fibrillin‐1 is a key constituent of extracellular microfibrils that affects the flexibility and tensile strength of the tissue, which are essential for heart function [22, 23]. Decorin expression is typically increased after AMI, and its deficiency has been shown to result in abnormal scar tissue formation and ventricular dilatation and dysfunction [24]. The decreased expression of fibrillin and decorin in the *Col15a1* ^*−/−*^ scars may impair the strength and elasticity of the scar area. As the patient prognosis after an infarction is determined not only by the size but also by the location, composition, structure and mechanical properties of the scar, the observed changes may lead to reduced stretching resistance and affect long‐term survival after AMI. In this study, long‐term follow‐up of mice was not performed, making it a relevant aspect for future studies.

Moreover, a deformed scar border zone can result in an increased risk of life‐threatening ventricular arrhythmias [25]. We observed that *Col15a1* ^*−/−*^ mice have significantly more immature than mature collagen fibres in the infarct border zone compared with WT mice. A higher ratio of mature collagen fibres in the infarct border zone has been associated with improved cardiac function following AMI [26], because mature fibres are more robust and provide more support to the injured myocardium.

Proper formation and remodelling of the remote myocardium is of utmost importance after AMI. As some changes in the ECM of the *Col15a1* ^*−/−*^ mice were observed already in the baseline, it is probable that the constitutive knockout compounds the effects during the development, thereby affecting partly the observed phenotype after AMI. In the remote LV, outside the scar area, the abnormal accumulation of collagen changes the myocardium morphology and diminishes the contractility of myocytes, finally leading to heart failure [5]. *Col15a1* ^*−/−*^ mice showed more severe pathological LV remodelling after AMI compared with the WT mice, including morphological ventricle modifications, different cardiomyocyte characteristics and increased tissue stiffness and fibrosis. This was accompanied by changes in the expression, composition and physical features of the ECM components, for example, by upregulation of fibrosis‐related genes *Loxl3, Col1a1* and *Col3a1* [27, 28]. The expression of the gene encoding lysyl‐3‐oxidase, a primary collagen cross‐linking enzyme in the heart associated with fibrosis and upregulated after AMI [29], was significantly higher in the remote areas of the *Col15a1* ^*−/−*^ mice than in the wild‐type mice. Accordingly, the expression levels of collagen types I and III were upregulated in the *Col15a1* ^*−/−*^ mice, whereas *Tgf‐β* was expressed similarly in both genotypes. We observed more proliferating cells and significantly more myofibroblasts in the remote areas of *Col15a1* ^*−/−*^ hearts compared with WT hearts. In addition, isolated primary fibroblast cultures from *Col15a1* ^*−/−*^ mice showed higher numbers of dividing cells than those derived from WT mice; these results suggest that ColXV may affect the proliferative activity of myo/fibroblasts and that its lack could thereby facilitate tissue fibrosis. ColXV has been identified in different fibroblast populations in several studies; for example, a recent study integrating single‐cell transcriptomic data identified the *Col15a1*‐positive fibroblast cluster as one of the two universal fibroblast subtypes across tissues [30]. Interestingly, downregulation of ColXV has been reported in fibroblasts derived from patients with Dupuytren's contracture, a fibroproliferative disorder characterised by abnormal proliferation of fibroblasts [13]. In spite of increased fibrosis in the remote myocardium of *Col15a1* ^*−/−*^ hearts, we did not observe a difference in the infarct scar size in the absence of ColXV. This difference is most probably due to different and highly unclear mechanisms between reparative and reactive fibrosis [5, 6, 31]. Besides that, the limited effect on the scar size can be due to a strong endothelial cell loss in the scar area; as based on the qPCR results, this cell type is the main producer of ColXV. Better understanding of the mechanisms underlying the progression of post‐AMI fibrosis in infarcted and noninfarcted areas, and exploring the characteristics of fibroblasts in the noninfarcted myocardium, would be needed for a deeper understanding of pathological remodelling after AMI.

In addition, the echocardiographic data indicate that, 1 week after AMI, ventricular dilatation was accelerated and stronger in the *Col15a1* ^*−/−*^ mice than in the WT ones. The EF and FS parameters were lower in the *Col15a1* ^*−/−*^ mice at 1‐ and 5‐week time points, indicating a more significant loss of LV systolic function and a significantly decreased pumping capacity compared with WT mice. The prolonged isovolumetric relaxation time in the *Col15a1* ^*−/−*^ mice suggests impaired LV relaxation at the end of the experiment [32]. The baseline echocardiographic data of *Col15a1* ^*−/−*^ male mice indicated a different cardiac phenotype compared with previously studied female mice [15]. This result supports the complex phenomenon of observed sex‐specific differences in cardiovascular diseases [33], such as improved survival and functional recovery of female mice after AMI [34].

Rearrangement of the ECM in response to MI changes myocyte morphology and intracellular architecture, contributing to contractile dysfunction [35, 36, 37]. Stiffer matrix can disrupt sarcomere assembly, resulting in different cardiomyocyte function by affecting cellular contraction, and causes aberrant gene expression that alters the balance between cardiac and ECM gene regulation [37]. Our observations indicated that these changes were stronger when ColXV was lacking. In addition to an altered ECM gene expression and stiffer matrix, we observed sarcomeric imbalance and different myocyte morphology in the *Col15a1* ^*−/−*^ mice compared with WT mice. We showed that after AMI, the distance between the Z‐lines was longer in *Col15a1* ^*−/−*^ mice than in WT littermates. Additionally, the α‐actinin signal was uneven. We also observed an increased signal for desmin, an intermediate filament and a sarcomeric linker protein in the remote area. The increase in the desmin signal in the *Col15a1* ^*−/−*^ mice could compensate for the unstable sarcomere structure, as suggested previously in the failing heart [38]. Furthermore, immunofluorescent analysis and qPCR indicated a broad expression change in the sarcomeric proteins between genotypes. Together, these findings suggest that changes in the ECM in the *Col15a1* ^*−/−*^ mice increases heart stiffness, leading to changes in cardiomyocyte morphology, gene expression and sarcolemmal instability after AMI. The finding of more adhesive cardiomyocytes and increased detachment after adding recombinant collagen XV is contradictory to the increased LV dilatation observed in the *Col15a1* ^*−/−*^ hearts after AMI. The LV dilatation is a result of myocyte slippage from the underlying matrix and a major risk factor for LV rupture [3, 4]. In this regard, analysing mechanosensitive and ECM adhesion markers, such as integrins and dystroglycan receptors, would be needed for resolving the possible compensatory mechanisms affecting the adhesive properties of cardiomyocytes in the absence of collagen XV.

To conclude, the cardiac dysfunction that we observed in *Col15a1* ^*−/−*^ hearts after AMI is due to a combination of changes in the scar area, infarct border zone and remote myocardium. The results show that a lack of ColXV causes aberrant cardiac remodelling after AMI, resulting in accelerated heart failure development. Although not affecting the scar size, the absence of ColXV changes scar structure, scar border zone and cardiac stiffness. The widespread effects are conducted through overall disrupted cardiac tissue and cardiomyocyte support because of increased fibrosis, improperly organised connective tissue, aberrant gene expression and changes in the intracellular cardiomyocyte architecture, leading to contractile and mechanical dysfunctions. Evidently, ColXV creates a biomechanically advantageous environment after AMI and is indicated to have a cardioprotective effect, probably affecting the long‐term survival after AMI. The increased ColXV expression in human heart infarcts may signify such cardiac protection. These actions support a crucial role for ColXV in maintaining myocardial structure and function in situations that challenge cardiac homeostasis.

## Materials and methods

### Human tissue materials and licences

Human tissue material consisted of formalin‐fixed paraffin‐embedded samples containing acute myocardial infarction (AMI) scars (*n* = 50) and average age‐ (54–77 years of age; mean age 62) and gender‐matched [men (80%) and women (20%)] heart tissue from individuals who died from noncardiac causes (*n* = 10) from the Finnish Genetic Study for Arrhythmic Events (FinGesture) study (University of Oulu), which has collected data and histological samples on autopsy‐verified sudden cardiac death victims from northern Finland since 1998. The medicolegal autopsies were performed in the Forensic Medicine Unit, Finnish Institute for Health and Welfare, Oulu, Finland, and at the Department of Forensic Medicine, University of Oulu, Oulu, Finland, during 1998–2017. Samples were taken from the lateral wall and from the infarct scar area. Of these samples, technically well‐stained samples (AMI, *n* = 20 and controls, *n* = 5) were included in the study. Both study groups included diagnoses of hypertension (40%) and type 2 diabetes (40%). Ethical permission was obtained from Northern Ostrobothnia Hospital District Ethical Committee (2222/05.07.00/2011). Permissions to use medicolegal autopsy material and gather data from the documents of medicolegal cause‐of‐death investigation were obtained from the Finnish Institute for Health and Welfare (Document record numbers: THL/873/5.05.00/2023 and THL/2529/6.02.00/2025). The work was carried out in compliance with Finnish law that regulates the donation, procurement, processing, storage and distribution of human organs, tissues and cells for medical purposes (Act on the Medical Use of Human Organs, Tissues and Cells; 101/2001). The studies were carried out following the provisions of the Helsinki Declaration (1983).

### *Col15a1* ^*−/−*^ mouse line and animal licences

The *Col15a1* ^*−/−*^ (*Col15a1* ^*tm1Pih*^, MGI:2386162) mouse line has been created in house. The generation and characterisation of the line has been published in 2001 by Eklund *et al*. [14]. For the current study, mice were backcrossed for at least 10 generations into the C57BL/6J OlaHsd (Harlan, Indianapolis, IN, USA). Animal experiments were conducted in the Laboratory Animal Centre of the University of Oulu (OULAC), following the 3R principles and national and international legislation and guidelines for laboratory animal experimentation.  Animal licences for the experiments have been granted by the Regional State Administrative Agency for Southern Finland (ESAVI) (licence numbers: ESAVI‐2010‐09664/Ym23, ESAVI/381/04.10.07/2014, ESAVI/7250/04.10.07/2014 and ESAVI/8134/04.10.07/2017) and by the Laboratory Animal Center of the Oulu University (internal licences 015/2011, 19/2014 OH1, 59/2014 OH1, 47/2014 and 30/2017). Experiments were approved by the Animal Care and Use Committee of the University of Oulu (Oulu, Finland). The animals were housed in individual cages at constant humidity (40%) and a 12‐h light–dark cycle (06:00–18:00 light, 18:00–06:00 dark) in a pathogen‐free environment and fed with standard chow. Male mice and age‐ and gender‐matched wild‐type (WT) controls were used for experiments.

### Isolation and culture of cardiac cells

Adult ventricular myocytes were isolated from 6‐month‐old WT and *Col15a1* ^*−/−*^ mice as described previously [39]. Briefly, the mice were deeply anaesthetised using 2% isoflurane inhalation, their hearts were rapidly excised, and their aortas were cannulated and perfused with HEPES‐buffered Tyrode's solution supplemented with 0.1% collagenase type II (Worthington, Lakewood, NJ, USA) and 2,3‐butanedione‐monoxime. The ventricular tissue was homogenised and myocytes were collected via low‐speed centrifugation (180 ***g*** for 1 min). The supernatant was collected and centrifuged at 300 ***g*** for 5 min to collect fibroblasts and endothelial cells; these cells were in PBS, 2‐mm EDTA, and 2% FBS and filtered through a 70‐μm nylon mesh (BD Falcon, Franklin Lakes, NJ, USA). The endothelial cells were isolated by immunomagnetic cell separation with a rat anti‐mouse CD31 antibody (BD Biosciences, Franklin Lakes, New Jersey, USA, 751862, 1 : 100) and anti‐rat IgG microbeads (Miltenyi Biotec, Bergisch Gladbach, Germany). For further use, cells were frozen at −70 °C.

Fibroblasts were plated after isolation onto uncoated wells in DMEM/F12 (Invitrogen, Waltham, MA, USA) supplemented with 10% FBS, 2 mm l‐glutamine and penicillin–streptomycin and incubated at 37 °C with 5% CO_2_. The cells were incubated for 72 h, and the medium was changed daily. Morphology was evaluated 6, 24 and 72 h after plating, and dividing fibroblasts were counted from 20× microscopic fields 6 and 24 h after plating. Cardiomyocytes were plated after isolation with a density of 10 000 cells/1.8 cm^2^ onto laminin‐coated wells (chamber slide) and incubated at 37 °C with 2% CO_2_ in a minimum essential medium (αMEM) supplemented with 5% FBS, insulin‐transferrin‐selenium (Invitrogen), 10 mm 2,3‐butanedione‐monoxime, 2 mm l‐glutamine and penicillin–streptomycin (Sigma‐Aldrich, Saint Louis, MO, USA). To study the effect of ColXV on cell adhesion, purified recombinant ColXV [40] was used to coat culture plates with or without laminin at a concentration of 10 μg·mL^−1^. After 2 h of incubation, nonattached cells were gently removed by changing the medium. After 6 h of plating, the total numbers of viable cardiomyocytes attached to laminin and laminin/ColXV were quantified under 10× microscopic fields (4 mice/genotype, 5 fields/mouse) to calculate the average number of attached cells as an indication of cardiomyocyte adherence. The widths and lengths of the cardiomyocytes were measured from a single line drawn along each cardiomyocyte using ImageJ. All values are expressed as the mean ± SD. All experiments were performed with mycoplasma‐free cells, which were routinely tested using PCR analysis.

### Myocardial infarction model

Eighteen‐ to twenty‐four‐month‐old collagen XV null male mice (*Col15a1* ^*−/−*^) (*n* = 7) and age‐ and gender‐matched WT controls (*n* = 7) were exposed to AMI through the permanent ligation of the left anterior descending coronary artery (LAD) according to a previously described protocol [41]. Besides, age‐ and gender‐matched collagen XV null (*n* = 6) and WT mice (*n* = 5) were subjected to a sham‐control operation without the ligation of LAD. For preoperative analgesia, mice were treated with a subcutaneous injection of carprofen (Rimadyl 5 mg·kg^−1^ s.c.) and buprenorphine (Temgesic, 0.05–0.1 mg·kg^−1^ s.c.). Thirty minutes later, they were anaesthetised with 2% isoflurane inhalation. Under deep anaesthesia, the rib cage of the mice was exposed, and an incision was made in the fourth intercostal space to expose the anterior heart surface. The heart was briefly removed from the thoracic cavity through this incision to reach the LAD coronary artery. A 6–0 silk suture thread was used to place an obstructing knot around the top margin of the LAD. The mouse was then allowed to breathe room air and monitored during the recovery period. The control animals were sham‐operated similarly, but the thread was only slid behind the LAD artery, and knots were not made. Postoperative analgesia (carprofen once per day, buprenorphine twice per day) was administered for 3 days, and the mice were hydrated with glucose (50 mg·mL^−1^) solution when needed. The animals were kept in a warmed environment (+25 °C) for the first 24 h after the operation, and food and water were served *ad libitum*. The survival after the operations between the genotypes was equal.

### Preparation of tissue samples for light and electron microscopy, quantitative real‐time PCR and elasticity measurements

The animals described in Myocardial infarction model were euthanised; the hearts were quickly removed and weighed, and the atria and right ventricle were separated. The left ventricle was weighed and cut into three horizontal layers. The top layer was embedded in Immu‐Mount® gel and fixed in a dry ice container. Samples for light and electron microscopy, qPCR and stiffness measurements were collected from the middle and lower layers (the apex), and pieces of infarct scar and healthy tissue were obtained for transmission electron microscopy (TEM) analysis.

### Tissue elasticity measurement

Tissue sections of left ventricles (LVs) (2 mm slices) were prepared from *Col15a1* ^−/−^ and WT mice (*n* = 4 mice per group) and mounted with glue on the bottom of a well filled with 0.9% NaCl solution. Stiffness measurements were conducted at room temperature between 0.5 and 3 h after dissecting the heart. The measurements were done from mid myocardium in the remote area (not from the fibrotic scar area). The elastic moduli were measured using a microprobe indenter device [42], as described before [15]. Briefly, a tensiometer probe (Kibron, Helsinki, Finland) with a 200 μm diameter flat‐bottomed needle was mounted on a 3‐D micromanipulator with a 160 nm step size (Eppendorf, Hamburg, Germany) attached to a ZeissAxiovert (Carl Zeiss, Jena, Germany) microscope. The probe was calibrated using the known surface tension of a pure water/air interface, and the stress applied to the probe as it was pushed onto the tissue was measured as a function of indentation depth. Three measurements per sample (3–4 per mouse) were made, and the stiffness coefficient was calculated with Microsoft Excel® software using the equation: (Vdiff * gravity)/(surface tension of water * calcoeff) where Vdiff (mV) is the voltage difference set when calibrating the probe and calcoeff is the calibration coefficient (mV·mg^−1^) originating from the probe calibration. Surface tension of water is 72.8 mN·m^−1^ and gravity is 9.8 m·s^−2^. From this formula, the stiffness coefficient was calculated as follows: (3247 mV * 9.8 m·s^−2^)/(72.8 mN·m^−1^) * 274.92 mV·mg^−1^ = 31 820.6/20 014.176 = 1.5899030767.

### Immunostaining

#### Immunohistochemical staining

Immunolabelling of human 5‐μm formalin‐fixed and paraffin‐embedded heart sections was carried out using the peroxidase‐based EnVision kit (Agilent Dako, Glostrup, Denmark) following the manufacturer's protocol. Immu‐Mount® (Thermo Scientific, Waltham, MA, USA) was used for mounting. All sections were counterstained with haematoxylin. A primary antibody against collagen XV (Sigma‐Aldrich HPA017913, 1 : 500) was used. Before staining, epitope retrieval was performed using a heat‐mediated method for 15 min with Tris/EDTA buffer (pH 9). For negative controls, the primary antibodies were omitted and replaced with phosphate‐buffered saline. Sections were imaged with a Leica DM LB2 microscope with a digital camera (DFC 320, Leica, Wetzlar, Germany). Staining area as a percentage of the whole tissue area was analysed semiquantitatively and classified according to their staining level into categories low (< 30%), medium (between 30% and 60%) and high (over 60%).

The mouse tissues were fixed in 4% formaldehyde and embedded in paraffin. Haematoxylin and eosin (HE) stained sections were used to analyse leukocyte infiltration, oedemic changes and focal necrotic areas in the cardiac tissue and to analyse cardiomyocyte and myocardial morphology. The immediate scar area and the remote myocardium were analysed separately. The mean thickness value of cardiomyocytes was assessed by measuring their diameters from cross‐sections (cardiomyocytes of 10 40× microscopic fields from three mice per group were measured). Sections were stained with Masson trichrome and picrosirius red (Direct Red 80; Sigma‐Aldrich) using standard protocols to visualise collagen and examine the fibrotic areas of the heart. Briefly, 5 μm thick PFA‐fixed sections were dewaxed in xylene and rehydrated with a decreasing ethanol series. For Masson staining, the sections were treated with nuclear stains celestin blue (Sigma‐Aldrich) and Harris' haematoxylin (Sigma‐Aldrich), followed by treatments with acid fuchsin (Sigma‐Aldrich) and phosphomolybdic acid (Sigma‐Aldrich). Finally, the sections were treated with methyl blue stain (Sigma‐Aldrich). For picrosirius red staining, the sections were treated with 0.2% phosphomolybdic acid for 5 min, followed by staining with 0.1% Direct Red 80/Sirius Red F3B (Sigma‐Aldrich) in saturated picric acid for 1 h at room temperature (RT). Both protocols were finalised by dehydration with an ethanol series, treatment with xylene and mounted with Pertex (Sigma‐Aldrich). The Masson's trichrome‐stained sections were imaged with a Leica DM LB2 microscope with a digital camera (DFC 320, Leica) and picrosirius red‐stained sections under bright and polarised light with an Olympus BX51 microscope and digital camera (DP71; Olympus, Tokyo, Japan). The scar size was determined by calculating the ratio between the scar area and the whole tissue area in the mid ventricle cross‐section.

A terminal deoxynucleotidyltransferase‐mediated dUTP nick end‐labelling (TUNEL) assay was performed using the *In Situ* Cell Death Detection Kit (Boehringer Mannheim, Mannheim, Germany) according to the manufacturer's protocol to detect apoptotic cells. The percentage of apoptotic cells from the total cell count was calculated. Endothelial cells were visualised using a CD31 monoclonal antibody (BD Pharmingen, Franklin Lakes, NJ, USA, 558736, 1 : 300) with a tyramide signal amplification (TSA) kit protocol (PerkinElmer, Waltham, MA, USA), followed by incubation with a biotinylated secondary anti‐rat antibody (Vector Laboratories, Newark, CA, USA, 1 : 300) at room temperature. Counterstaining was performed with haematoxylin. A Leica DM LB2 microscope with a digital camera (DFC 320, Leica) was used for imaging, and pictures were processed using leica im50 software. ImageJ software was used to quantify and analyse apoptotic cells (TUNEL assay) and the capillary number (CD31 staining) in 10 separate sections (0.06 mm^2^) from the hearts (three per genotype) and to quantify cardiac fibrosis in Masson trichrome‐stained sections (five to seven mice/genotype). Qualitative analysis of collagen content was done by viewing picrosirius red‐stained tissue sections under linear polarised light (Olympus BX51 microscope), where thicker fibres were visualised in red and thinner fibres in green. Images were obtained with a colour digital camera (DP71; Olympus) and processed to RGB for automated thresholding using the ImageJ analysis software. The red and green areas were used to calculate the ratios between differentially stained collagen fibres.

#### Immunofluorescence staining

Mouse tissue samples were analysed using a standard IF protocol for 5 μm cryosections cut from the top layers of the left ventricles with a Leica cryomicrotome. Briefly, sections were fixed with ice‐cold methanol for 1 h at 20 °C, washed with PBS, blocked with 50% FCS and 1% Triton‐X100 in PBS for 1 h, and incubated with a primary antibody overnight at 4 °C and with the secondary antibody for 1 h at room temperature. The primary anti‐mouse antibodies were as follows: collagen XV (83AA, 1 : 500) [8], laminin (Enzo Life Sciences, Seattle, WA, USA, ALX‐804‐190, alpha 2 chain, 1 : 200), α‐SMA (Sigma‐Aldrich C6198, Cy3‐conjugated, 1 : 200), Ki‐67 (Abcam, Campridge, UK, 15580, 1 : 1000), Cd45 (BD Biosciences 553084, 1 : 100), Cd31 (BD Biosciences 550274, 1 : 300), α‐actinin (Sigma‐Aldrich A7811, sarcomeric, 1 : 500), fibrillin‐1 (1 : 200) [43], dystrophin (Abcam ab15277, 1 : 100), decorin (Abcam ab175404, 1 : 200), desmin (Sigma‐Aldrich D8281, 1 : 20), connexin 43 (Sigma‐Aldrich C6219, 1 : 300), desmoplakin (Santa Cruz, Dallas, TX, USA, sc‐33 555, 1 : 20), gelsolin (Abcam ab74420, 1 : 200) and tropoelastin (Abcam ab21600, 1 : 100). Anti‐mouse, anti‐rat or anti‐rabbit Cy2 or Cy3 (Jackson Immunoresearch, West Grove, PA, USA, 1 : 300), Alexa Fluor 488 (Invitrogen) or Alexa Fluor 647 (Jackson Immunoresearch) were used as secondary antibodies. Cell nuclei were detected with DAPI (Sigma‐Aldrich). The stained sections were visualised under a fluorescence microscope (Olympus FluoView FV1000 or Leica SP8 Falcon confocal microscope) and analysed using fluoview viewer (Olympus) or the las x (Leica) software. The imagej software was used to quantify signal areas positive for different antibodies on fluorescent images.

#### Immunocytochemistry

Isolated adult ventricular myocytes from WT and *Col15a1* ^*−/−*^ mice were seeded and allowed to attach, as described in the chapter above. The cells were fixed with a mixture of methanol/acetone (70%/30%) and blocked with 10% goat serum in PBS and incubated with anti‐dystrophin antibody (Abcam ab15277, 1 : 100). Myofibrils were stained red with Alexa Fluor 568 Phalloidin (Invitrogen, 1 : 300), and cell nuclei were detected with DAPI (Sigma‐Aldrich).

### Transmission electron microscopy

Heart samples were fixed in a 1% glutaraldehyde/4% formaldehyde mixture in 0.1 m phosphate buffer (pH 7.4) for 12 h at room temperature; postfixed in 1% osmium tetroxide; dehydrated in acetone; and embedded in Epon LX 112 (Ladd Research Industries, Essex Junction, VT, USA). Semi‐thin sections (500 nm) were stained with toluidine blue for area selection, and thin sections (70 nm) were cut with a Leica Ultracut UCT ultramicrotome (FC6; Leica), stained in uranyl acetate and lead citrate, and examined in a Tecnai G2 Spirit 120 kV transmission electron microscope (FEI Europe, Eindhoven, Netherlands). Images were captured with a Quemesa CCD camera and analysed using iTEM software (Olympus Soft Imaging Solutions GMBH).

### Echocardiography

Cardiac dimensions and function were analysed under isoflurane anaesthesia (4% induction, 1.5% maintenance) by transthoracic echocardiography. Cardiac parameters were measured using a VisualSonics (Toronto, Canada) Vevo 2100 high‐resolution ultrasound imaging system equipped with an MS‐550 D linear array transducer (40 MHz, axial res: 40 μm, lateral res: 90 μm). Echo was performed 1 week before the LAD ligation and one and 5 weeks after the operation. Short axis (SAX) and parasternal long axis (PSLA) views were used for image acquisition. On the SAX B‐mode image (Fig. 9), systolic and diastolic endocardial areas were measured, and fractional area change (FAC) was calculated using the following equation: FAC = [((Area;d – Area;s)/Area;d) × 100]. On SAX stand M‐mode image, left ventricular internal diameter in systole and diastole (LVID;s and LVID;d), interventricular septum (IVS) thickness, left ventricular posterior wall thickness in systole and diastole (LVPW;s and LVPW;d) were measured, and LV fractional shortening (FS) and ejection fraction (EF) were calculated from conventional line‐to‐borders and LV‐trace protocols as follows: FS (%) = [(LVID;d‐LVID;s)/LVID;d] × 100 and EF(%) = [(LV Vol;d − LV Vol;s)/LV Vol;d] × 100 where LV Vol = [7/(2,4 + LVID)] × LVID^3^. Formulas are taken from Vevo Lab v.5.8.1 ultrasound desktop calculating software. Representative M‐mode images demonstrate the measurement methods (Fig. 9). Transmitral flow velocity was recorded with pulse wave Doppler in apical four‐chamber view to evaluate left ventricular diastolic function. The peak flow velocities of the early diastolic filling wave (E) and the late diastolic filling wave (A) were measured, and the E/A ratio was calculated. Three measurements of consecutive cardiac cycles were taken for each variable. Measurements were performed by a skilled sonographer who was blinded to the treatments or genotype. After the last echocardiography, the animals were sacrificed, and tissue samples were collected for further analysis.

### RNA extraction and quantitative real‐time PCR

Mouse heart samples (*n* = 4–6/group) were quickly dissected at sacrifice and frozen in liquid nitrogen. According to the manufacturer's instructions, total RNA from the tissue samples and from frozen cardiac cell fractions (cardiomyocytes, fibroblasts and endothelial cells) was extracted using the RNeasy Mini Kit (QIAGEN, Hilden, Germany). cDNA was produced with the iScript cDNA Synthesis Kit (Bio‐Rad, Hercules, CA, USA). RT‐qPCR analyses were performed in duplicate using iTaq SYBR Green Supermix (Bio‐Rad) and a Bio‐Rad CFX96 real‐time PCR detection system. Results were analysed with the cfx manager 3.1 Software (Bio‐Rad), and relative quantification was performed using the ΔΔ*C*_t_ method using *Gapdh and Actb* as reference genes. Primer sequences used for qPCR are presented in Table S1. A previously published primer was used for *Col15a1* [15].

### Statistical analysis

The differences between two groups were analysed using an unpaired Student's two‐tailed *t*‐test. Sample sets containing several samples from the same biological origin were analysed with a nested *t*‐test or nested one‐way ANOVA followed by Tukey's HSD (alpha = 0.05). Boxplots are presented as the median, 25th and 75th percentiles, with whiskers indicating maximal and minimal values. Normal distribution was assessed using the Kolmogorov–Smirnov test and homogeneity of variance with Levene's test or *F*‐test. Chi‐squared and Fisher's exact test were used to assess the difference between cardiomyocyte aspect ratio proportions. *P*‐values < 0.05 were considered statistically significant (Symbols in figures: *P < 0.05; **P < 0.01; ***P < 0.001; ****P < 0.0001). The data are presented as the means ± standard deviation (SD). Statistical analyses were performed using ibm spss statistics software v. 22.0 (SPSS Inc., Chicago, IL, USA) or graphpad prism 10 software (GraphPad Software, San Diego, CA, USA).

## Author contributions

S‐MK, MA, LE, KR, LYS, JM, RK and TP contributed to study design and/or conception. HH, LP and JJ contributed to the acquisition of data for the work. S‐MK, MA, ZS, JM, LV, EG, PJ and IM performed experiments, and analysed and interpreted data. S‐MK and MA drafted the manuscript. All authors reviewed the manuscript for important intellectual content.

## Conflict of interest

The authors declare no conflict of interest.

## Supporting information


**Table S1.** Primer sequences used for qPCR.

## Data Availability

The data that support the findings of this study are available from the corresponding author (taina.pihlajaniemi@oulu.fi) upon reasonable request.
