# Supplementary material for: Collagen XV preserves heart function and protects from pathological remodelling after myocardial infarction
Source: FEBS J. 2025 Aug 20;293(2):418–42. doi: 10.1111/febs.70212 (PMC12820609; doi:10.1111/febs.70212)
Supplement: Supplementary file 1 — Table S1. Primer sequences used for qPCR. [file FEBS-293-418-s001.pdf]

Karppinen S-M et al.

Collagen XV preserves heart function and protects from pathological remodelling after myocardial infarction

Supporting Information

Online Supplementary Table

Supplementary Table 1. Primer sequences used for qPCR.

Gene	Forward primer 5'->3'	Reverse primer 5'->3'
Actb	AGAGGGAAATCGTGCGTGAC	CAATAGTGATGACCTGGCCGT
Col1a1	TGTGTGCGATGACGTGCAAT	GGGTCCCTCGACTCCTACA
Col3a1	CCACGAGGTGACAAAGGTGA	GCCAGGGAATCCTCGATGT
Col15a1	TGTGTCCTTTACCACCGGCT	GTTGGCACCAGGCCCA
Gapdh	AACTTTGGCATTGTGGAAGG	GGATGCAGGGATGATGTTCT
Gsn	CTGAGCACACAGCTGGACTG	AAGCTGGTGGTACCTGATGC
Loxl1	GAGTGCATATTGCGCTTCCC	GGTTGCCGAAGTCACAGGT
Loxl2	CAGAGAAGACCTACAACCCCA	AGTGCCCGTGCAGTTCATAG
Loxl3	TGTGACAGAATGCGCCTCTC	ACCTCAATGACGTTGGAGTCT
Myh6	GGTGCCAAGAAGATGCACG	TTATGTTTATTGTGGATTGGCCACAG
Nppa	GAAAAGCAAACTGAGGGCTCTG	CCTACCCCCGAAGCAGCT
Nppb	AGGCGAGACAAGGGAGAACA	GGAGATCCATGCCGCAGA
Tgfb1	CCGCAACAACGCCATCTATG	CTCTGCACGGGACAGCAAT
Tek	ATGTGGAAGTCGAGAGGCGAT	CGAATAGCCATCCACTATTGTCC
Ttn	GCCGTGGTAGAAGGTTTCCA	CTACAATTGTGGCTGGTTCTAGG
Vcl	CGGGTTGGAAAAGAGACTGT	GGAACCGAGTATGGGTCTGA
